# Low-Velocity Impact Response of Biaxial Glass-Fiber Fabric Composites: Influence of Fabric Layer Number Under Progressively Increasing Impact Energy

**DOI:** 10.3390/polym18192412

**Published:** 2026-10-02

**Authors:** George Cătălin Cristea, Lorena Deleanu, Maria Gherghișan, Mario Constandache, Claudiu Marian Martin, Ioana Gabriela Chiracu, Cătălin Pîrvu, Alina Dragomirescu

**Affiliations:** 1 National Institute for Aerospace Research (INCAS) “Elie Carafoli”, 220 Iuliu Maniu, 061126 Bucharest, Romania; cristea.george@incas.ro (G.C.C.); pirvu.catalin@incas.ro (C.P.); dragomirescu.alina@incas.ro (A.D.); 2 Technical Sciences Academy of Romania (ASTR), 26 Dacia, 010413 Bucharest, Romania; deleanu.lorena18@gmail.com; 3 National University of Science and Technology “Politehnica” Bucharest, Faculty of Mechanics and Mecatronics, Department of Machine Element and Tribology, 313 Splaiul Independentei, District 6, 060042 Bucharest, Romania; mario.constandache@gmail.com (M.C.); claudiumartin398@gmail.com (C.M.M.); chiracu.gabriela@gmail.com (I.G.C.); 4 “Ovidius” University, 124 Mamaia Avenue, 900527 Constanța, Romania

**Keywords:** glass fiber biaxial fabric, layers, composite, low-velocity impact, maximum force, maximum displacement, absorbed energy, transition from partial penetration to complete perforation

## Abstract

Glass-fiber-reinforced polymer composites are of interest for lightweight structural applications, including marine structures, where accidental low-velocity impact is an important design consideration. This study investigates the low-velocity impact response of polyester-matrix composites reinforced with 4 layers, 6 layers, and 8 layers of biaxial glass fabric. A progressive test program, designed according to the number of fabric layers, used increasing impact-energy levels to cover both an indentation-dominated non-perforating regime and complete perforation, with the transition toward complete perforation in each configuration guiding the energy range selected for the configuration with the next higher number of fabric layers. Instrumented drop-weight tests with a 16 mm hemispherical impactor were analyzed using force, displacement, velocity, and energy histories. At the highest fully non-perforating energy levels of 30 J, 60 J, and 100 J, maximum contact forces were 7.25 kN, 13.05 kN, and 17.45 kN, while absorbed energies were approximately 28.7 J, 54.1 J, and 95.3 J. At 40 J, 80 J, and 120 J, respectively, the composite series entered the transition regime, with complete perforation observed for some of the tested specimens. The investigated parameters showed different diagnostic values: some were sensitive to the impactor mass–velocity combination, whereas displacement, energy, and velocity histories more clearly reflected the change in impact regime. The progressive test campaign, extended up to complete perforation, provides a practical means of identifying the transition between impact regimes and establishing application-specific impact limits. For design purposes, components exposed to accidental impact should therefore incorporate an adequate margin below the experimentally identified transition energy range.

## 1. Introduction

Long-fiber-reinforced polymer composites (for example, composites containing carbon or glass fibers embedded in a polymer matrix) are increasingly used due to their high strength-to-weight ratio. However, their impact behavior is complex and differs significantly from that of metallic materials.

Recent studies on biaxial, non-crimp, woven, and hybrid fiber-reinforced polymer composites have reported valuable data on tensile [1,2], flexural [3,4], combined tensile and flexural [5,6,7], and low-velocity impact behavior [8,9,10]. However, the experimental descriptions are not always complete from the point of view of reproducibility and cross-comparison. In several cases, relevant parameters such as composite thickness, fiber volume or mass fraction, impactor geometry, impact velocity, and the relation between imposed energy and penetration/perforation regime are only partially reported. Therefore, a comparative discussion based not only on mechanical response but also on material architecture and impact-test configuration is useful for understanding the layer-number- and architecture-dependent behavior of composites reinforced with biaxial glass-fiber fabric.

Priya I.I.M. and Senthil R. [1] investigated 2 mm-thick biaxial E-glass/epoxy NCF (non-crimp fabric) laminates with ±45° and ±60° fiber orientations using tensile tests according to ASTM D3518 on 250 mm × 25 mm specimens, reporting an average maximum axial stress of 176.82 MPa for the ±45° configuration.

Libonati F. and Vergani L.M. [2] investigated unidirectional and ±45° non-crimp E-glass/epoxy laminates under static tensile loading and reported a tensile strength of approximately 142 ± 3 MPa for the ±45° configuration, while also relating the mechanical response to damage evolution monitored by thermography and microscopic observations.

Ullah S. et al. [11] manufactured composites made of 6 layers of biaxial E-glass fabric with an areal density of 450 g/m^2^ and a vinyl ester resin with a tensile strength of 69 MPa and strain at break of 3.2%. The fiber orientation of each of the six layers was 0°, 15°, 30°, 45°, 60°, and 75° and the authors carried out tensile tests (sample dimensions 200 mm × 25 mm), three-point bending tests (sample of 120 mm × 13 mm) and Charpy tests (samples of 100 mm × 10 mm without notch). The results point out that the higher values of the tensile strength were obtained when one of the fiber directions was aligned with the loading one. The influence of fiber orientation on the impact strength of the biaxial composites is particularly strong for the 15° and 30° orientations. The highest flexural strength and modulus were obtained at 0°, 30°, and 45°. The study points out that loading type, fiber orientation towards loading and stacking sequence influence the experimental results and the mechanical parameters did not follow the same ranking for these three different loading tests.

Constantinescu G. et al. [5] used biaxial non-crimp-fabric composites, with carbon/epoxy, glass/epoxy, and flax/bioepoxy components, under tensile and flexural loading, taking into account ISO 527-4 and ISO 14125 [12,13], for small electric-vehicle chassis applications, highlighting the interest in biaxial fabrics and different types of resin.

Shi E. and Montesano J. [14] tested a biaxial arrangement of a unidirectional fabric by stacking the sequence [0°/90°]s, comparing the response of the composites at ambient and low temperatures, under tensile loading.

Similarly, Yu L. et al. [7] reported that biaxial warp-knitted glass/epoxy composites exhibited higher tensile and bending strengths than comparable plain-woven laminates.

Zhang et al. [8] investigated the low-velocity impact response of carbon/glass non-crimp fabric composites, including a glass-fiber reference laminate, at drop-weight impact energies of 20 J, 30 J, and 40 J; the analysis combined force–displacement response, peak force, absorbed energy, and C-scan damage assessment, showing that, at the lowest energy level, the induced damage could remain difficult to identify by visual inspection alone.

Boukar A. et al. [9] investigated the low-velocity impact response of biaxial NCF glass/polyester laminates, including a [±45°]_3s_ configuration, using force–time and energy–time histories together with high-speed imaging and digital image correlation. Although their edge-on/bending impact configuration differs from the centrally impacted plates considered in the present study, their results provide relevant information on the dynamic response and damage development of biaxial NCF glass-fiber composites.

Dalfi H. K. et al. [10] investigated eight-ply non-crimp cross-ply epoxy laminates with a balanced [0°/90°]_4_ stacking sequence, reinforced either with S-glass yarns or with hybrid S-glass/polypropylene yarns, at impact energies of 15 J, 25 J, 35 J, and 50 J using an Instron CEAST 9350 drop-weight system with a 20 mm impactor. Their analysis included force–time and force–displacement histories, peak force, peak deformation, elastic and absorbed energies, contact duration, and damage degree, complemented by ultrasonic C-scan and microscopic examination of the impacted laminates. The energy–time histories exhibited a maximum value, E_max_, followed by a decrease to a final absorbed-energy level, E_a_; the difference between these values was interpreted as residual or rebound energy, reflecting the partial return of stored energy after impactor arrest. The authors also related changes in the force response and energy dissipation to progressive damage mechanisms such as matrix cracking, delamination, fiber fracture, and, for the hybrid laminates, inter- and intra-yarn cracking.

Three-dimensional woven reinforcements represent an alternative to conventional multilayer laminates because through-thickness yarns can improve delamination resistance and impact damage tolerance. However, these benefits are accompanied by a more complex reinforcement architecture and manufacturing route, possible reductions in some in-plane properties due to yarn crimp, and higher production costs. Consequently, 3D woven composites may be advantageous for applications requiring enhanced through-thickness performance, but they are not necessarily a preferable solution for large-area structures where cost, manufacturability, and scalability are important design constraints [15].

The present study extends this type of interpretation by following the response over progressively increasing impact-energy ranges selected according to the number of fabric layers, up to the transition from rebound-dominated partial penetration to complete perforation.

Gupta A.K. et al. [16] fabricated GFRP composites with sequences of [0°, 90°]s, [−45°, +45°]s and [−60°, 0°, 60°]s, with s = 2, s being the number of repeated set of layers, and an epoxy resin. Sample dimensions were 150 mm × 100 mm. The impact energy was set at 15 J. The unidirectional E-glass fabric had an areal density of 500 g/m^2^. Specimens used for tensile testing had dimensions 250 × 15 × 1 mm^3^. Samples for compressive strength determination were rectangular strips with dimensions 140 × 10 × 2 mm^3^. Similarly, shear strength and shear modulus were determined using specimens of dimensions 120 × 30 × 5 mm^3^. The interlaminar shear strength of the composites was determined by a short-beam shear test using 36 mm × 12 mm × 6 mm specimens subjected to three-point bending. However, no information on the composite thickness was provided, and the specimens used for tensile, shear, and bending tests had different dimensions, making it difficult to compare the composite behavior under different loading conditions because specimen dimensions may strongly influence the measured response.

Studies using progressive impact-energy levels or penetration/perforation threshold approaches in composite, polymeric and metallic plates are reported in order to identify transitions between damage regimes and determine critical impact-energy levels [17,18,19,20,21,22]. They provide data useful for assessing collision-related structural risks and the resistance of components under accidental impact loading.

Reddy T.S. et al. [23] tested laminates made of a biaxial fabric ([0°/90°] and 0.25 mm thickness) and epoxy resin, using hemispherical steel impactor of 16 mm diameter with impact energies in the rage of 50–150 J (in order to reach the perforation threshold), and with laminate thicknesses of 5 mm, 7 mm and 10 mm, and a fiber volume fraction of 0.62. Tests carried out at three conditioning temperatures (−20 °C, 25 °C, and 100 °C) could be relevant to structures exposed to a wider temperature range during exploitation, particularly marine and wind-energy structures. Statistical analysis of the results (based on three repeated tests under the same conditions) has shown that the composite thickness has the highest influence on peak force. Also, thickness increased the perforation threshold and changed the damage mode.

Evci C. [24] reported the behavior of composites made of plain-weave and unidirectional (arranged [0°/90°]_s_) E-glass fiber fabrics with areal densities of 800 g/m^2^ and 500 g/m^2^, respectively. The thicknesses of composites varied from 2 mm to 8 mm. A spherical tup insert of 10 mm diameter was used as the striker. This author also showed that three distinct zones characterizing the impact damage behavior of composites can be distinguished from typical load–displacement curves: rebound, penetration, and perforation.

The comparison also highlights a recurrent limitation in the available literature: some key parameters required for a rigorous reproduction of low-velocity impact tests are not consistently reported. Missing information commonly concerns the impactor nose diameter, impact velocity, exact composite thickness, or fiber volume fraction. These omissions make direct comparison difficult, especially when composites differ simultaneously in fiber architecture, matrix type, stacking sequence, and thickness.

While progressive impact testing and thickness-related effects have been investigated in previous GFRP studies, the present work focuses on the combined interpretation of multiple instrumented impact parameters across configuration-specific energy ranges extending to the transition toward complete perforation. The comparison highlights which parameters primarily reflect the imposed test conditions and which provide useful information for distinguishing successive impact-response regimes.

The investigated composites were manufactured using the same biaxial ±45° non-crimp glass-fiber fabric and an unsaturated polyester resin, with three configurations containing 4, 6, and 8 fabric layers. For each configuration, an impact-energy range was selected to cover non-perforating responses and the transition toward complete perforation. The main objective was to evaluate these responses through the time histories of force, displacement, absorbed energy, and impactor velocity, complemented by macroscopic examination of the resulting damage and failure features. The proposed approach is intended to support the assessment and design of lightweight composite components, including those potentially intended for marine applications, where accidental low-velocity impact may occur, by identifying characteristic response regimes and configuration-specific transition intervals toward complete perforation.

The proposed approach may support the assessment and design of lightweight composite components potentially intended for marine applications, while their performance under relevant environmental conditions requires further investigation.

## 2. Materials and Methods

The present impact campaign was designed according to the number of fabric layers and used progressively increasing impact-energy levels, conceptually related to energy-profiling approaches used to identify penetration and perforation thresholds in composites. The orientation of the biaxial fabric was kept unchanged between successive layers for all three composite configurations; no additional rotation of individual layers was introduced.

Unlike studies performed at a single impact energy, the progressive increase in impact energy makes it possible to follow the transition from light indentation and rebound through partial penetration to complete perforation, while also showing how the transition toward complete perforation shifts across configurations with increasing fabric layer number and resulting laminate thickness.

In the present study, partial penetration denotes local advancement of the impactor into the laminate while the specimen is still able to arrest it, whereas complete perforation denotes through-thickness passage of the impactor through the composite.

The reinforcement, a biaxial ±45° non-crimp glass fiber fabric, having an areal density of 620 g/m^2^ (Dipex spol. s.r.o., Sered, Slovakia) [25], is less frequently reported in the literature than conventional woven or unidirectional reinforcements. Fabric sheets of 300 mm × 300 mm were cut and weighed, to prepare sets of 4 layers, 6 layers and 8 layers.

After a literature survey on resins for glass fiber composites [26,27,28], the resin Enydyne H 68372 TA, together with the Metox–50W from the range provided by Rompolimer Composites (Bucharest, Romania), was selected [27,29]. Enydyne H 68372 TA is an unsaturated polyester resin with an orthophthalic DCPD (dicyclopentadiene) formulation, intended for high-performance applications. The reported properties of the resin include a density of 1100 kg/m^3^, a tensile strength of 45 MPa, a tensile strain at break of 1.5%, a flexural strength of 65 MPa and a flexural modulus of 3.3 GPa. Metox–50W, based on 50% MEKP (methyl ethyl ketone peroxide), was added at 1.5 wt.% relative to the resin mass (1.5 g per 100 g of resin). For a 100 g resin batch, the reported gel time is 19–27 min, with a maximum reaction temperature below 135 °C.

Laboratory processing was used to manufacture composite plates potentially intended for marine applications. The mold (with an active area of 300 mm × 300 mm) was treated with Cirex CP-10 (produced by Airétec, Amiens, France, and supplied by RomPolimer Composites SA, Bucharest, Romania) [30], a release wax, to form a thin monomolecular barrier. The fabric sheets were manually impregnated, and the composite was consolidated under a uniform pressure of 0.5–0.7 MPa for 8–12 h, demolded, and stored under laboratory conditions at 20–25 °C for approximately 10 days before impact testing. The set of specimens prepared for impact testing was manufactured from composite plates reinforced with biaxial glass-fiber fabric in three different composite configurations: 4s, 6s, and 8s. These designations correspond to specimens manufactured with 4 layers, 6 layers, and 8 layers of biaxial fabric, respectively.

Beyond coupon- or plate-level characterization, low-velocity impact testing has also been extended to geometrically tailored structural components. For example, Chen S. et al. [31] investigated the axial impact response of thin-walled A6061 tubes with periodically distributed internal and external grooves, combining drop-weight experiments with numerical simulations. A similar transition from flat specimens to component-level testing would represent a logical next step for the composite configurations investigated here.

Impact tests were performed using an Instron CEAST 9350 (ITW Test & Measurement Italia S.r.l.—Instron CEAST Division, Pianezza, Torino, Italy) impact-testing machine at the National Institute for Aerospace Research “Elie Carafoli”—INCAS, Bucharest, Romania. For each composite configuration, several impact energy levels were selected progressively in order to highlight the transition from local damage and partial penetration to complete perforation. The experimental setup is shown in Figure 1, including the overall impact-testing system, the impactor–specimen region, and the specimen support and clamping arrangement.

ISO 6603-2:2023 [32] was used as a starting point for defining the instrumented impact-test configuration, while the specimen geometry, impact-energy ranges, and set of analyzed parameters were adapted to the objectives of the present study. Square specimens with nominal in-plane dimensions of approximately 100 mm × 100 mm were tested using a 40 mm support ring and the same clamping arrangement throughout the experimental program. The specimen dimensions therefore differed from the 60 mm square geometry associated with the 40 mm support ring in ISO 6603-2:2023 [32]; however, the effective unsupported impact area and the boundary conditions were maintained constant for all tests. Small variations in the external specimen dimensions resulting from cutting occurred outside the active test region. A 16 mm hemispherical impactor was used throughout the experimental program. This diameter differs from the preferred 20 mm and alternative 10 mm striker diameters specified in ISO 6603-2:2023 and should therefore be considered when comparing the present results with standardized puncture-impact data. The hemispherical geometry itself is consistent with the general puncture-impact configuration described in the standard.

For each composite configuration and impact-energy level, five replicate specimens were tested. This number corresponds to the minimum number of specimens specified in ISO 6603-2:2023 [32] for tests performed under constant conditions, whereas ten specimens are required only for arbitration purposes. The use of five replicates at each impact-energy level provided a consistent basis for assessing repeatability and specimen-to-specimen variability throughout the progressive impact campaign. In total, 60 instrumented impact tests were performed.

For each composite configuration, the mass and thickness characteristics were determined from the 20 specimens subsequently used in the impact tests. The mean specimen mass was calculated as

(1) $$m_{c,\mathrm{set}}=\frac{1}{N}\sum_{i=1}^{N}m_{c,i}$$

where *N* = 20 is the number of specimens for each composite configuration. The theoretical mass of the biaxial glass-fiber fabric contained in each specimen was calculated from the measured specimen area, the number of fabric layers in the composite, and the fabric areal density, *ρ*_areal_ = 620 g/m^2^:

*m_f_*_,*i*_ = *A_i_ρ_areal_n_s_* (2)

where *n_s_* is the number of fabric layers (4, 6, or 8). The corresponding average theoretical fabric mass was then calculated as

(3) $$m_{f,\mathrm{set}}=\frac{1}{N}\sum_{i=1}^{N}m_{f,i}$$

The resin mass was estimated for each specimen as the difference between the measured composite mass and the theoretical fabric mass,

*m_r_*_,*i*_ = *m_c_*_,*i*_ − *m_f_*_,*i*_ (4)

and the average resin mass was obtained from the 20 specimens of each configuration. The fiber mass ratio was calculated for each specimen as

(5) $$R_{\mathrm{mf}/c,i}=\frac{m_{f,i}}{m_{c,i}}$$

where *m_f_*_,*i*_ is the theoretical mass of the biaxial glass-fiber fabric in specimen *i*. The fiber mass ratio was subsequently averaged for each composite configuration.

Specimen thickness was measured at four locations near corners for each specimen. The average thickness of each specimen, *h_i_*, was calculated as

(6) $$h_{i}=\frac{h_{i1}+h_{i2}+h_{i3}+h_{i4}}{4}$$

and the average thickness of each composite configuration was obtained from the 20 individual specimen values. Table 1 summarizes the resulting average values and standard deviations. It presents the average mass and thickness for the tested specimens and also the fiber mass ratio.

As expected, increasing the number of fabric layers resulted in a substantial increase in specimen mass. Compared with the 4s configuration, the average specimen mass increased by 68.1% for 6s and by 138.5% for 8s. While the theoretical fabric mass increased approximately in proportion to the number of reinforcement layers, the calculated resin mass increased at a higher rate. Consequently, the fiber mass ratio decreased moderately from 0.68 ± 0.04 for 4s to 0.61 ± 0.06 for 6s and 0.59 ± 0.07 for 8s. These values remain within the range reported for structural glass-fiber/polymer composites, for which fiber mass fractions of approximately 0.60 or higher are commonly reported, depending on reinforcement architecture and manufacturing route [33,34,35]. The gradual decrease in fiber mass ratio with increasing composite thickness suggests that the thicker composites contained a larger proportion of resin under the selected processing conditions. Since the same consolidation pressure was applied to all composite configurations, this tendency also suggests that the consolidation conditions may need to be adjusted as the number of fabric layers increases when a more uniform fiber-to-matrix ratio is required.

The coefficients of variation (CVs) in Table 1 provide a relative measure of specimen-to-specimen variability. The CV of specimen mass increased from 6.41% for the 4s composite to 11.30% and 11.49% for the 6s and 8s composites, respectively. In contrast, thickness variability remained relatively similar across the configurations, with CV values of 10.81%, 11.38%, and 8.91%, respectively. The calculated resin mass exhibited higher relative variability (CV = 19.57–26.64%), which may be associated with differences in resin distribution and consolidation during manual manufacturing. The removal of excess cured resin during edge finishing may also have contributed to specimen mass variability. These manufacturing-related differences may influence the local stiffness and impact response, particularly within the transition region toward complete perforation. However, their individual contributions to the observed scatter in impact parameters were not quantified.

In addition to the calculated fiber mass ratio, the approximate constituent volume fractions were estimated from the corresponding mass fractions and nominal constituent densities. Because the void content was not measured independently, the calculated values should be regarded as two-phase estimates of the fiber and matrix volume fractions rather than experimentally determined constituent volume fractions.

Since the specific glass-fiber composition was not provided in the supplier documentation, the fiber density was not known exactly. According to Wallenberger F. T. et al. [36,37], boron-containing E-glass has a density of approximately 2.55 g/cm^3^, whereas boron-free E-glass has a slightly higher density of approximately 2.62 g/cm^3^. Therefore, the fiber volume fraction was estimated using this literature-based density range. The resulting estimated *V_f_* ranges were approximately 0.472–0.478, 0.396–0.403, and 0.377–0.383 for the 4s, 6s, and 8s composites, respectively. These values should be regarded as estimates rather than experimentally measured constituent volume fractions.

The average specimen thickness increased from 2.22 ± 0.24 mm for 4s to 3.78 ± 0.43 mm for 6s and 5.39 ± 0.48 mm for 8s, confirming the expected progressive increase in composite thickness with the number of reinforcement layers.

The fiber mass ratios obtained for the present composites, ranging from 0.59 ± 0.07 to 0.68 ± 0.04, are comparable to values reported for structural glass-fiber/polyester composites. Varga C. et al. [33] reported a glass-fiber content of 61.4 ± 2.1 wt.% for woven E-glass/polyester composites, while Aranha R. et al. [34] obtained 59.70 ± 3.27 wt.% for compression-molded glass-fiber/polyester composites. Of particular relevance to potential marine applications, Han Z. et al. [35] reported an experimentally determined glass-fiber content of 61.86 wt.% for a high-fiber-content GFRP yacht hull composite. These comparisons indicate that the reinforcement content of the investigated composites is within the range used for structural GFRP composites.

The 4s configuration, with a fiber mass ratio of 0.68 ± 0.04, lies toward the upper part of this range, whereas the 6s and 8s configurations remain close to the values reported for structural and marine GFRP composites.

For each impact-energy level, five specimens were tested to assess the repeatability of the results and to obtain representative average values for the analyzed impact parameters. Table 2 presents the tests carried out for this study. The red cells indicate the transition energy levels at which complete perforation was first observed for at least one specimen in each set of five tests. The blue cells are for tested specimen sets for which only the partial penetration was noticed.

The specimens were coded including the number of biaxial fabric layers: 4s for composite specimens with four layers, 6s for composite specimens with six layers, and 8s for composite specimens with eight layers.

It should be noted that the selected impact-energy levels were obtained by adjusting both the moving mass and the drop height and, consequently, the impact velocity. Such combinations of drop height and moving mass are also employed in low-velocity impact studies using the Instron CEAST 9350 to extend the attainable impact-energy range [38,39]. Therefore, impact energy was used as the primary test parameter for organizing the test campaign. The measured response is discussed in relation to the specified impact-energy conditions and the corresponding mass–velocity combinations reported in Table 2.

Table 3 provides the corresponding test conditions for each energy level. Two moving-mass configurations, 5.277 kg and 15.277 kg, were used during the experimental campaign. The heavier configuration was required for the higher impact-energy levels because of the maximum available drop height of the testing system. Consequently, comparisons between conditions obtained using different moving masses reflect the combined influence of impact energy and the corresponding mass–velocity combination.

Figure 2 shows one plate for each composite configuration, marked for cutting into square specimens.

The main specifications of the Instron CEAST 9350 impact testing machine [40] are as follows: drop height range of the impact system, 30–1100 mm; impactor mass range, 1–3.5 kg for the light impactor, 3–22 kg for the standard impactor, and 3–37 kg for the standard impactor with additional plates; impact velocity range, 0.77–4.64 m/s; impact energy range, 0.3–37 J for light impact, 0.89–237 J for standard impact, and 0.89–400 J for standard impact with additional plates; and temperature range, −50–100 °C, adjustable in 1 °C increments. The specimens were clamped using the pneumatic fixture of the impact-testing system, which applied a pressure of 0.3 MPa over a circular clamping sector surrounding the impact region. The circular opening had a diameter of 40 mm. The specimens were therefore circumferentially clamped and not simply supported, and the same clamping configuration was maintained for all impact tests.

For these impact tests, an Instron impact head, code C-7529-322, mounted on an Instron CEAST 9350 testing system, was used [41]. Its function is to apply impact loading to the specimen surface and to enable recording of the mechanical response of the material during contact between the impactor and the specimen. The impact head, with a hardness of 60–62 HRC, was fitted with a hemispherical insert with a diameter of 16 mm. The hemispherical geometry of the impactor provides a controlled localized loading of the specimen and is commonly used in instrumented impact testing of polymeric materials and fiber-reinforced composites. The use of an instrumented impactor allows the contact force to be recorded and the displacement, velocity, and absorbed energy to be determined during the test.

The tests were performed under dry contact conditions. Preliminary trials with a lubricated impactor resulted in composite fragments adhering to its surface. Therefore, dry contact was selected and maintained throughout the experimental campaign. This condition differs from the lubrication procedure specified in ISO 6603-2:2023 and should be considered when comparing the present results with standardized puncture-impact data.

For each set of five replicate tests, the moving mass and drop height were kept constant. The small variations in measured impact velocity and actual impact energy therefore represent test-to-test variability. Two moving-mass configurations, 5.277 kg and 15.277 kg, were used because the higher impact-energy levels could not be reached with the lighter configuration within the maximum available drop height of the testing system.

Figure 3 provides a general macroscopic view of the progressive damage developed in the 4s specimens as the impact energy increased from 10 J to 40 J. At 10 J, the impacted region remains relatively limited and the visible damage is limited, consisting mainly of a shallow indentation and local changes in surface appearance.

With increasing impact energy, the indentation becomes progressively more pronounced and visible damage to the reinforcement develops, including yarn distortion, partial yarn breakage and, at the higher energy levels, complete rupture of individual yarns or fiber bundles. Some specimens tested at 40 J clearly exhibit a damage morphology associated with complete perforation of the composite. However, the opening remaining after removal of the specimen from the test fixture may be considerably smaller than the 16 mm impactor diameter. This smaller residual opening may be explained, at least in part, by the relatively long yarn and fiber-bundle segments surrounding the perforated region. Even when locally fractured, these segments remain connected to the surrounding composite and can partially recover and reposition after removal of the impactor. In the fractured region, the ends of broken yarns appear locally separated from the surrounding matrix or retain only small matrix fragments, reducing their local constraint and allowing additional repositioning after unloading. Consequently, the dimensions of the post-impact opening should not be interpreted directly as either the impactor diameter or the maximum opening developed during its passage through the composite.

The photographs also illustrate the limitations of a purely macroscopic post-impact assessment. Even fractured reinforcement may partially return toward its initial position after unloading and thereby mask the maximum extent of the temporary opening produced during penetration. Macroscopic images should therefore be interpreted together with knowledge of the deformation and failure behavior of the reinforcement and, where possible, with the instrumented impact response. The whitish regions surrounding the impact zone provide additional qualitative evidence of damage, potentially involving the matrix and fiber–matrix interfaces. Such changes in appearance may be associated with matrix cracking and fragmentation, local fiber–matrix debonding, and localized or more extended delamination. However, the individual contribution of these mechanisms cannot be distinguished unambiguously from macroscopic photographs alone.

Figure 4 presents the response of five specimens of the 4s composite tested under nominally identical conditions at an impact energy of 40 J. The good overlap of the curves during the initial stage of impact is consistent with repeatable initial loading conditions and a similar early response among the specimens.

For force, absorbed energy, and displacement, two response regions can generally be distinguished. During the first region, extending approximately up to the vicinity of the maximum value of the respective parameter, the curves remain closely grouped. Beyond this stage, the responses progressively diverge, producing a more pronounced fan-out of the curves. This increased scatter is consistent with the growing influence of progressive local damage and specimen-specific response, rather than necessarily indicating poor repeatability of the imposed test conditions. Such damage may involve matrix cracking, delamination, local crushing, and yarn or fiber-bundle breakage, as commonly reported for impacted laminated composites. However, the occurrence and relative contribution of these individual mechanisms were not directly resolved in the present study. Consequently, specimen-to-specimen variability becomes more evident during the later stages of the impact event. This two-region response, characterized by a relatively reproducible early stage followed by increased scatter as damage develops, is commonly observed in polymeric and composite materials, where the post-peak response becomes increasingly governed by localized and specimen-specific failure mechanisms. At 40 J, this divergence is particularly relevant because the 4s composite is tested at its transition energy level, where partial penetration and complete perforation coexist among the five specimens. The same tendency is further confirmed by the parameter plots discussed in the Results and Discussion sections.

## 3. Results

### 3.1. Impact Test Parameters

Several studies have adopted multi-parameter approaches to characterize the low-velocity impact response of GFRP composites, combining quantities such as peak force, displacement, absorbed energy, contact duration, damage extent, and penetration-related parameters to assess the evolution of impact damage and the influence of composite configuration or thickness [23,42]. A particularly comprehensive analysis was proposed by Khashaba U. A. [43] for woven GFRP composites with a [0°/±45°/90°]s stacking sequence, in which more than twenty impact-assessment parameters were grouped and correlated with impact-induced damage over an energy range of 2–60 J. The study also distinguished several energy regions and identified thresholds associated with damage initiation, penetration initiation, full penetration, and perforation.

Related multi-parameter approaches have also been employed in previous studies by some of the present authors. Cristea G. C. et al. [44] investigated the low-velocity impact response of glass-fiber composites manufactured with different resin systems and reinforcement architectures, considering maximum force, absorbed energy, displacement, and damage characteristics. Păduraru I. et al. [45] analyzed the influence of impact energy and composite thickness on several impact characteristics, including maximum force, time to maximum force, and impact duration. These studies indicated that the combined interpretation of several complementary parameters can provide a more detailed description of the impact response than individual peak quantities considered separately.

The present study extends this approach to a progressive impact campaign involving composites with different fabric layer numbers and resulting thicknesses and examines not only the magnitude of the analyzed impact parameters, but also their different sensitivities and diagnostic value in distinguishing successive damage regimes up to complete perforation. The characteristic impact parameters depend on the impact regime, i.e., whether partial penetration or complete perforation occurs. Some parameters can be measured or calculated for both regimes, such as the maximum force, F_max_, and the time corresponding to the maximum force, t_(Fmax)_, whereas others do not have a meaningful physical interpretation under both conditions. For example, in the present analysis, impact duration was determined only for tests involving partial penetration (without complete perforation). The parameters extracted from the instrumented impact tests were grouped into force-related, energy-related, and kinematic quantities (Figure 5). Their physical interpretation depends on the impact regime, particularly on whether the impactor is arrested by the composite or complete perforation occurs.

- Force-related parameters (Figure 5a).

The maximum force, F_max_, is the peak contact force recorded during the impact event and characterizes the maximum load transmitted to the composite specimen. The initial impact time, t_0_, is operationally defined as the time corresponding to the last zero-force value preceding the significant increase associated with impactor–specimen contact. For impacts in which the impactor is arrested, the final contact time, t_f_, is defined as the time corresponding to the first zero-force value after the force peak. The contact duration is therefore calculated as

∆*t* = *t_f_* − *t*_0_ (7)

The time to maximum force, t_(Fmax)_, represents the interval between contact initiation and attainment of F_max_, while d_(Fmax)_ is the impactor displacement at the time of maximum force.

The quantities t_(Ff=0)_ and d_(Ff=0)_ characterize the time and displacement at which the contact force returns to zero. These latter parameters have a consistent physical meaning only when complete perforation does not occur, because, during complete perforation, a residual low force may persist while the impactor continues to interact with the damaged material.

- Energy-related parameters (Figure 5b).

In the present study, E_max_ denotes the maximum energy value recorded during the impact event, as identified from the energy–time history. For impacts followed by impactor arrest and rebound, Emax corresponds to a distinct maximum of the curve and occurs approximately when the impactor velocity reaches zero. At this stage, the initial kinetic energy of the impactor, originating from the potential energy imposed through the selected moving mass and drop height, has been transferred predominantly to the specimen as deformation energy and energy dissipated through irreversible damage. During rebound, part of the elastically stored energy is returned to the impactor, and the energy curve consequently decreases. Thus, for these tests, t_(Emax)_ ≃ t_(v=0)_, with small differences being possible because the quantities are obtained from different measurement or calculation channels. In the case of complete perforation, however, the energy–time history may continue to increase without developing a distinct local maximum; in such cases, Emax represents the highest energy value recorded within the analyzed impact interval. Therefore, the physical interpretation of Emax depends on the impact regime. The final absorbed energy, E_absorbed_, represents the net energy transferred to the specimen by the end of the impact event. For complete perforation, this value may also include contributions associated with continued material failure, opening of the perforation path, and frictional interaction between the impactor and the damaged material.

- Kinematic parameters (Figure 5c,d).

– The initial impact velocity, v_0_, is the impactor velocity at the beginning of contact and characterizes one of the imposed test conditions. The velocity–time history describes impactor deceleration and, when complete perforation does not occur, arrest and rebound.
– The time t_(v=0)_ identifies the instant at which the impactor is stopped before its velocity changes sign. The maximum displacement, d_max_, is the largest impactor displacement reached before rebound. It should not be interpreted solely as elastic or irreversible deformation of the composite, since it reflects the combined effects of global bending and local indentation, potentially including compaction, crushing, matrix and fiber damage, and partial advancement of the impactor through damaged plies. When complete perforation occurs, the displacement continues to increase as the impactor passes through the composite; consequently, a comparable d_max_ associated with impactor arrest is no longer defined.

The analysis of a broader set of impact parameters, as adopted in the present study, contributes to a more comprehensive understanding of impact as a progressive damage process and helps distinguish the different components of the measured response. Such a multi-parameter approach may therefore support the selection of relevant impact-response parameters for the assessment and design of composites, rather than relying exclusively on a limited number of conventionally reported parameters.

The analysis of a broader set of impact parameters, as adopted in the present study, contributes to a more comprehensive understanding of impact as a progressive damage process and helps identify the different sensitivities of the measured response quantities to impact severity and test conditions. This distinction is relevant for engineering applications, since some parameters may be more suitable for design-oriented assessment, whereas others may provide useful indicators for in situ monitoring of impact-induced structural response and damage evolution. Therefore, composite design and assessment may benefit from considering a complementary set of impact parameters rather than relying exclusively on a limited number of conventionally reported quantities.

The parameters presented in Figure 6, Figure 7, Figure 8 and Figure 9 must be interpreted according to the impact regime. The maximum force, time to maximum force, effective impact energy, and absorbed energy can be reported for all impact energy levels, including cases involving complete perforation. In contrast, parameters such as the time at which the force returns to zero, displacement when the force returns to zero, maximum displacement in the rebound regime, and time at zero velocity have the defined physical significance only for impacts in which the impactor is arrested without complete perforation. The time histories in Figure 6, Figure 7, Figure 8 and Figure 9 include all four energy levels; summary plots of arrest-defined parameters include only the non-perforating levels.

The impact parameters were analyzed at the energy levels selected for each composite configuration, taking into account the corresponding impact regime.

### 3.2. Analysis of the Impact Parameters on Parameter—Time Graphics

Parameters defined by the return of the force to zero, impactor stopping, zero velocity, or maximum displacement before rebound have physical significance only for impacts in which the impactor is arrested without complete perforation. In the case of complete perforation, the impactor continues to move through the composite. These parameters therefore no longer characterize impactor arrest; any residual force reflects continued interaction with the damaged material. Consequently, these parameters were not plotted at the transition energy level of each composite configuration.

For Figure 6, Figure 7, Figure 8 and Figure 9, the results are arranged in a common graphical format to facilitate comparison. The three rows represent the 4-, 6-, and 8-layer composites, while the four columns correspond to progressively increasing impact-energy levels, from the lowest investigated level to the transition energy level. Each graph superimposes the five replicate tests, allowing comparison of the response evolution with impact energy, initial repeatability, and specimen-to-specimen variability.

The force–time histories (Figure 6) reveal three qualitatively distinct response regimes. At low impact severity, exemplified by the 4s composite at 10 J, the force curve is comparatively smooth and asymmetric bell-shaped, indicating deformation-dominated behavior without pronounced force drops associated with progressive loss of load-bearing capacity. Similar smooth force–time responses have been associated with non-perforating impacts, whereas increasingly irregular or serrated curves characterize more severe damage and penetration regimes [45,46]. Păduraru I. et al. [45], investigating glass-fiber/epoxy composites containing two, four, and six quadriaxial fabric layers, also reported marked changes in the force–time response as the impact regime evolved from partial penetration to complete perforation.

At intermediate energies, loading remains relatively smooth up to F_max_, while the post-peak response becomes increasingly serrated. Force drops and recoveries suggest progressive damage and load redistribution to intact regions. Possible mechanisms include matrix cracking, interfacial damage, and fiber-bundle breakage, although their contributions were not directly determined. Khashaba U. A. [43] reported similar post-peak force recovery due to load redistribution to neighboring intact fibers. Thus, post-peak unloading is not purely elastic, as damage and energy dissipation continue after F_max_.

For a given composite configuration, F_max_ becomes less sensitive to increasing impact energy as the transition energy level is approached. For the 4s composite, average Fmax increases from 5.204 kN at 10 J to 7.505 kN at 40 J (a factor of 1.44), but only by about 3.6% from 30 J (7.246 kN) to 40 J. For the 8s composite, Fmax remains within 17.378–18.184 kN over 80–120 J. Composites with more fabric layers develop higher contact forces, consistent with their greater thickness, bending stiffness, and load-bearing capacity, whereas the more compliant 4s composite shows a stronger evolution of Fmax before the transition level. Similar thickness effects on peak force and perforation resistance were reported by Reddy et al. [23] and Liu et al. [47,48].

The limited increase in F_max_ near perforation agrees with Liu et al. [47,48], who reported peak-force stabilization, and Khashaba U. A. [43], who observed an almost constant Fmax of 4.5 kN between 50 J and 60 J, with complete perforation at 60 J. This suggests a limiting load-bearing level, beyond which additional impact energy is increasingly accommodated by deformation and progressive damage, including perforation-path development, rather than a proportional increase in F_max_.

At complete perforation, the main force pulse becomes narrower and is followed by an extended low-force tail. This response is consistent with continued interaction between the impactor and the severely damaged composite during its passage through the specimen, potentially including friction against broken fibers and the edges of the perforated region. Khashaba U. A. [43] similarly associated the post-perforation response with friction between the hemispherical impactor and the perforated hole, together with continuing damage propagation. Wan Y. et al. [49], in recent experiments on thin woven GFRP composites extending to complete perforation, also showed that increasing impact severity progressively suppresses rebound and shifts the response toward extensive fiber breakage, tearing, delamination, and penetration.

At the highest energy investigated for each composite configuration, the five nominally identical tests do not always produce the same final regime: some specimens are completely perforated, whereas others arrest the impactor after severe partial penetration. This specimen-to-specimen variability indicates that the highest investigated energy level lies within a transition interval rather than representing a unique deterministic perforation threshold. Consequently, the complete force–time history has greater diagnostic value for distinguishing severe impact regimes than F_max_ alone. The evolution from a relatively smooth response, through increasingly serrated post-peak behavior, to a narrowed pulse followed by a low-force tail provides qualitative indicators of progressive damage and the transition toward complete perforation.

The absorbed energy–time histories (Figure 7) complement the force–time response and clearly distinguish the different impact regimes.

During the initial stage, the five replicate curves overlap closely, indicating good repeatability, whereas the scatter becomes more pronounced as the transferred energy approaches Emax and during the subsequent response, consistent with the increasing influence of progressive damage and specimen-to-specimen variability.

For indentation-dominated impacts and partial penetration, energy peaks and then decreases as stored energy is partly returned during rebound. Under complete perforation, the impactor is not arrested, and energy remains high as transfer continues during damage and perforation. Thus, final energy depends on the impact regime. At the transition energy level, both responses occur among the five replicates, indicating a transition interval rather than a unique threshold. Parameters defined only for impactor arrest must therefore be evaluated separately from complete-perforation responses.

The displacement–time histories (Figure 8) provide a direct indication of the impact regime. For indentation-dominated impacts and partial penetration, displacement increases to d_max_, where the impactor velocity becomes zero and the slope of the displacement–time curve vanishes. Rebound then produces a decrease in displacement.

In contrast, during complete perforation, the impactor retains a positive velocity and continues through the composite; therefore, the slope does not change sign and no displacement maximum associated with impactor arrest is observed.

The region around d_max_ is also mechanically significant because the impactor reaches its maximum displacement, while damage may continue to evolve. Consequently, the post-d_max_ response should not be interpreted as purely elastic recovery, even when rebound occurs.

The velocity–time histories (Figure 9) provide a direct kinematic distinction between partial penetration and complete perforation. For non-perforating impacts, the impactor decelerates to zero velocity and subsequently rebounds, producing a change in the sign of velocity. During complete perforation, the velocity decreases substantially but remains positive, indicating that the impactor is not arrested and continues through the composite. Thus, the change in sign of the impactor velocity provides a clear criterion for distinguishing rebound from complete perforation.

Taken together, the force–time, absorbed energy–time, displacement–time, and velocity–time histories provide a complementary description of the impact response and allow the transition between partial penetration and complete perforation to be identified more reliably than any single parameter considered alone. The close agreement among the five replicate tests, particularly during the initial stages of impact, is consistent with good repeatability of the initial loading conditions and a similar early mechanical response among the specimens.

### 3.3. Analysis of Values for the Impact Parameters

Figure 10 presents the average maximum force, F_max_, as a function of impact energy for the 4s, 6s, and 8s composites. Overall, higher F_max_ values are obtained for composites with a larger number of biaxial ±45° non-crimp glass-fiber fabric layers.

However, the effects associated with fabric layer number and the resulting composite thickness cannot be separated from those of the different impact-energy ranges because each composite configuration was tested over a different energy range selected to extend up to the transition toward complete perforation. The higher force levels measured for the 6s and 8s composites therefore reflect the combined influence of a larger number of fabric layers (with the resulting greater thickness) and the higher imposed impact energies.

Within a given composite configuration, F_max_ shows a reduced sensitivity to increasing impact energy as the transition energy level is approached. For the 4s composite, F_max_ increases from 5.204 kN at 10 J to 7.246 kN at 30 J and only to 7.505 kN at 40 J; thus, a 33% increase in impact energy from 30 to 40 J produces an increase of only about 3.6% in F_max_. The 6s response is non-monotonic, increasing from 10.748 kN at 40 J to 13.046 kN at 60 J and decreasing slightly to 12.310 kN at 80 J, while the 8s values remain within a narrow range of approximately 17.4–18.2 kN over 80–120 J. Similar reductions in the sensitivity of peak force near perforation have been reported by Liu D. et al. [47,48] and, more recently, by Khashaba U. A. [43].

These results indicate a tendency toward leveling off of F_max_, rather than a proportional increase with impact energy, as severe damage and perforation are approached. Additional impact energy is therefore increasingly accommodated by deformation and progressive damage, including the development of the perforation path, rather than producing a proportional increase in peak contact force. Consequently, F_max_ remains useful for characterizing the maximum load-bearing response, but it should not be used alone to identify the transition to complete perforation; this transition is more clearly revealed by the combined energy–time, displacement–time, and velocity–time histories.

Figure 11 presents the displacement at maximum force, d_(Fmax)_, for the 4s, 6s, and 8s composites. For each configuration, d_(Fmax)_ varies within a relatively narrow range and does not increase monotonically with impact energy. The values are approximately 4.9–5.7 mm for 4s, 5.8–7.2 mm for 6s, and 7.2–9.0 mm for 8s. Although larger values are generally obtained for composites with more fabric layers, this trend reflects differences in fabric layer number and the resulting composite thickness, together with the different impact-energy ranges.

Near the transition energy level, d_(Fmax)_ shows only limited variation and remains close to the values recorded at the immediately lower, non-perforating energy level. Together with the behavior of F_max_, this indicates that the maximum contact force and its corresponding displacement are reached before the later stages of damage evolution that may culminate in complete perforation. Consequently, d_(Fmax)_, like F_max_, has reduced sensitivity to the transition between partial penetration and complete perforation and should be interpreted together with the complete energy–time, displacement–time, and velocity–time histories.

At the transition energy level, t_(Fmax)_ decreases relative to the immediately preceding non-perforating energy level for all three composite configurations (Figure 12). Although the overall variation in t_(Fmax)_ with impact energy is not monotonic, this common tendency indicates that F_max_ is reached earlier at the transition energy level. This behavior may reflect the combined influence of the higher impact velocity and evolving damage, although their individual contributions cannot be separated in the present tests.

However, t_(Fmax)_ should not be considered an independent indicator of complete perforation and is better interpreted together with F_max_ and the displacement-, absorbed-energy-, and velocity-based responses.

The displacement d_(Ff=0)_ represents the impactor displacement when the contact force returns to zero and, for non-perforating impacts, provides an apparent measure of the residual deformation in the impacted region. It should not, however, be interpreted as plastic deformation alone, since it also reflects local damage and irreversible deformation. As shown in Figure 13, d_(Ff=0)_ increases markedly with impact energy for the 4s composite and more moderately for the 8s composite, whereas the 6s results vary only slightly, decreasing from 5.52 mm at 40 J to 5.17 mm at 60 J.

The time t_(Ff=0)_ marks the end of effective contact between the impactor and the specimen and is meaningful only for non-perforating impacts followed by impactor arrest and rebound (Figure 14). The variation in t_(Ff=0)_ with impact energy is non-monotonic, reflecting the combined influence of the imposed impactor mass–velocity combination and the spatially and temporally non-uniform development of damage during contact. At the transition energy level, t_(Ff=0)_ is not consistently defined because the return of the contact force to zero does not consistently represent the end of contact in tests involving complete perforation; therefore, these values were excluded. Consequently, t_(Ff=0)_ is primarily useful for characterizing the duration of non-perforating impacts rather than as an independent indicator of impact severity.

Figure 15 presents the average recorded maximum energy, E_max_, as a function of the nominal impact energy. The recorded values are generally close to the prescribed energy levels for all three composite configurations, and the small standard deviations confirm good repeatability of the imposed impact conditions. Thus, variations in the recorded maximum energy are generally limited; however, differences in the response parameters should also be interpreted in relation to the imposed impactor mass–velocity combinations. At the transition energy level, both partial penetration and complete perforation occurred among the five replicates; therefore, parameters whose physical meaning depends on the impact regime were not averaged across these mechanically different responses.

Figure 16 shows that d_(Emax)_ generally increases with impact energy within the non-perforating regime. The increase is more pronounced for the 4s composite, whereas the 6s and 8s composites exhibit a more gradual variation over their respective impact-energy ranges. This behavior is consistent with the greater structural compliance of the 4s composite. However, the influence of fabric layer number and the resulting composite thickness cannot be isolated from that of impact energy, as each composite configuration was tested over a different energy range.

Figure 17 shows that, within the non-perforating regime, t_(Emax)_ increases with impact energy for the 4s and 6s composites, whereas the 8s composite exhibits a slight decrease between 80 J and 90 J, followed by an increase at 100 J. For all three configurations, the highest values are reached at the upper non-perforating energy level. Moreover, t_(Emax)_ is consistently greater than t_(Fmax)_, indicating that energy transfer continues after the maximum contact force has been reached. Thus, t_(Emax)_ is useful for describing the temporal evolution of the impact event, but not as an independent indicator of the transition to complete perforation.

The absorbed energy, E_absorbed_, was compared only for non-perforating impacts (Figure 18), where it represents the net energy transferred to the composite after impactor rebound, including energy dissipated through irreversible deformation and damage. After complete perforation, the recorded energy also includes contributions associated with formation of the perforation path and continued interaction between the impactor and the damaged material. Therefore, values from the two regimes were not combined in the same comparison.

The maximum impactor velocity, v_max_, primarily characterizes the imposed test conditions (Figure 19). Because the selected impact energies were obtained using different combinations of moving mass and drop height, v_max_ does not vary uniquely with impact energy; within series using the same moving mass, however, it increases consistently with energy. The small standard deviations indicate good repeatability of the imposed impact conditions. Therefore, v_max_ is considered mainly a test-control parameter rather than an indicator of composite impact performance.

The time to zero impactor velocity, t_(v=0)_, identifies impactor arrest before rebound and is therefore defined only for non-perforating impacts (Figure 20). Its variation depends not only on the composite response and impact energy, but also on the moving mass–velocity combination used to generate that energy. This influence is particularly evident for the 6s composite, for which t_(v=0)_ increases from 3.35 ms at 40 J to 5.58 ms at 50 J and remains nearly constant at 60 J (5.68 ms). The marked increase between 40 J and 50 J coincides with a change from a lighter, faster impactor configuration to a heavier, slower one. Because the maximum drop height was limited by the impact-testing setup, the higher impact-energy levels were obtained by varying both drop height and moving mass. Consequently, impact energy does not necessarily correspond to a continuous increase in impact velocity when the moving mass changes between test conditions.

The maximum displacement, d_max_, increases monotonically with impact energy within the non-perforating regime for all three composite configurations (Figure 21). The increase is more pronounced for the thinner 4s composite, consistent with its greater structural compliance, whereas the thicker 6s and 8s composites show smaller increments. However, d_max_ represents the maximum impactor displacement at arrest, not the residual deformation of the composite. It reflects the combined effects of global bending, local indentation, irreversible deformation, and local penetration into the damaged composite.

Despite this combined physical origin, the systematic increase in d_max_ makes it a useful quantitative indicator of increasing impact severity before complete perforation. From a design perspective, a configuration-specific experimentally established allowable d_max_ could therefore provide a practical warning criterion for composite components subjected to accidental impact, including lightweight marine structures.

The experimental results show that all five specimens were able to arrest the impactor at 30 J, 60 J, and 100 J for the 4s, 6s, and 8s composites, respectively. At the next investigated energy levels of 40 J, 80 J, and 120 J, complete perforation occurred in 4 of 5, 4 of 5, and 3 of 5 specimens, respectively. Thus, the transition toward complete perforation occurred at progressively higher impact-energy ranges as the number of biaxial fabric layers, and consequently the composite thickness, increased. These results identify configuration-specific transition intervals rather than unique deterministic perforation thresholds. However, the effect of thickness cannot be isolated from that of the fiber mass ratio, which also varied among the manufactured configurations.

The observed differences in impact response reflect the combined characteristics of the manufactured configurations, including fabric layer number, resulting composite thickness, and fiber mass ratio; the individual contributions of these factors cannot be isolated from the present experimental design.

Figure 22 shows representative specimens tested at the lowest investigated impact-energy level for each composite configuration. The visible response is dominated by localized indentation on the impacted face and out-of-plane bending on the back face, without clear macroscopic fiber-bundle rupture or through-thickness penetration. The deformation remains concentrated around the impact region, while the surrounding material retains its apparent structural integrity. Differences between the impacted and back faces reflect the local contact loading and the associated bending response of the composite. At these energy levels, all specimens arrested the impactor, and the photographs are consistent with the non-perforating response identified from the instrumented histories. Although limited internal damage may already be present, its nature and extent cannot be determined reliably from macroscopic inspection alone.

Figure 23 shows the damage morphology at the highest non-perforating impact-energy level for each composite configuration. Compared with the specimens in Figure 22, the impacted faces exhibit markedly more severe localized damage, including deeper indentation, crushing, distortion, and visible breakage of glass-fiber yarns or bundles, while the back faces show greater out-of-plane deformation and local yarn failure.

The damaged regions are not necessarily symmetric, reflecting the heterogeneous local response of the fabric-reinforced composites. Although additional internal damage is likely present, it cannot be resolved from the macroscopic photographs alone. Despite the severe visible damage, the remaining load-bearing material is still able to arrest the impactor, corresponding to partial penetration without complete perforation.

Figure 24 shows the macroscopic damage associated with complete perforation. Compared with the non-perforating regime, the specimens exhibit severe localized indentation and crushing on the impacted face, together with yarn displacement and fiber-bundle breakage visible on both faces and a through-thickness perforation path. The residual opening may appear smaller than the 16 mm impactor diameter because of partial recovery and repositioning of damaged fiber bundles after unloading. Therefore, complete perforation was not identified from hole size alone, but was confirmed from the instrumented response, particularly by the absence of rebound and the continued increase in impactor displacement while its velocity remains positive, supported by the extended low-force tail after the force peak.

The present damage assessment is intentionally limited to macroscopic inspection, as the main objective of the study is to characterize the transition from partial penetration to complete perforation through the instrumented impact response. Internal damage mechanisms are therefore not directly resolved, and any discussion of matrix cracking, delamination, or fiber–matrix debonding is based on possible mechanisms consistent with the measured response and visible damage features, rather than on direct evidence from dedicated microstructural examination.

## 4. Diagnostic Assessment of Impact Parameters

The comparison of the individual response parameters shows that their usefulness cannot be assessed solely from the magnitude of their variation with impact energy. Some quantities primarily describe the imposed test conditions, whereas others reflect the structural response of the composite or provide clearer evidence of a change in the impact regime. Therefore, the parameters were qualitatively classified according to their sensitivity to the experimental configuration, their physical meaning, and their diagnostic value for distinguishing non-perforating impact from complete perforation.

The resulting assessment is summarized in Table 4 and is supported by the corresponding parameter–energy plots.

The diagnostic assessment in Table 4 should not be interpreted as defining universal numerical thresholds applicable to the entire family of composites. Quantitative limits are configuration-dependent and should be established separately for each composite architecture, taking into account the number of biaxial fabric layers, the resulting composite thickness, and the specified impact and boundary conditions.

At the highest investigated non-perforating impact-energy levels, the mean maximum displacement, d_max_, calculated as the average of five replicate tests, was 9.18 ± 1.00 mm for the 4s composite at 30 J, 8.99 ± 0.37 mm for the 6s composite at 60 J, and 11.02 ± 0.38 mm for the 8s composite at 100 J. These values characterize the maximum displacement reached by each composite configuration under its respective test conditions. They may serve as configuration-specific criteria for assessing the impact response, but they do not define the onset of complete perforation.

A more accurate characterization of the range of d_max_ values associated with the transition toward complete perforation would require additional tests at intermediate impact energies between the highest fully non-perforating level and the energy at which complete perforation was observed.

Quantitative post-impact metrics such as residual indentation depth, damage area, or crack length were not determined in the present campaign and would require dedicated profilometric or calibrated image-based measurements.

## 5. Conclusions

The present study demonstrated the usefulness of a progressive impact campaign with energy ranges selected according to the number of fabric layers for characterizing the low-velocity impact response of biaxial glass-fiber fabric composites. Rather than testing all composite configurations at a single impact-energy level, the investigated energy range was progressively increased as the number of fabric layers increased, allowing successive response regimes to be identified, from indentation-dominated deformation to partial penetration without complete perforation and, finally, complete perforation. This approach provides a more informative characterization of a family of composites because it identifies not only individual response values but also the evolution and transition between impact regimes. The highest investigated energy levels, 40 J, 80 J, and 120 J for the 4s, 6s, and 8s composites, respectively, lay within configuration-specific transition intervals in which both impactor arrest and complete perforation were observed among nominally identical specimens.

A second important outcome of the study is the combined interpretation of a relatively broad set of force-, energy-, displacement-, velocity-, and time-related parameters. The results showed that these quantities have different sensitivities to impact severity and to the imposed test configuration. Maximum force, F_max_, provides useful information on the load-bearing response but shows reduced sensitivity to further increases in impact energy as the transition energy level is approached and therefore cannot identify complete perforation by itself. In contrast, the complete displacement–time, velocity–time, and absorbed energy–time histories provide clearer evidence of impactor arrest, rebound, and the transition to complete perforation. Maximum displacement, d_max_, also showed a consistent increase with impact severity within the non-perforating regime and may therefore provide a useful design-oriented indicator when a configuration-specific allowable displacement can be established experimentally.

The manufactured composites also exhibited systematic changes in thickness and calculated fiber mass ratio with increasing fabric layer number. The average thickness increased from 2.22 ± 0.24 mm for 4s to 3.78 ± 0.43 mm for 6s and 5.39 ± 0.48 mm for 8s, while the calculated fiber mass ratio ranged from 0.59 ± 0.07 to 0.68 ± 0.04. The calculated fiber mass ratios indicate that the investigated manufacturing route produced composites with a substantial reinforcement content. The moderate decrease in fiber mass ratio with increasing fabric layer number also suggests that consolidation conditions warrant further investigation if a more uniform fiber-to-matrix ratio is desired across composite configurations.

The present results are specific to the investigated biaxial ±45° glass-fiber fabric composites and to the applied impact and boundary conditions. Because the impact energy was increased in discrete levels, the transition toward complete perforation is bounded by configuration-specific energy intervals rather than defined by a single critical energy value. The study was intentionally focused on the global instrumented impact response and on the diagnostic value of the measured force-, displacement-, velocity-, energy-, and time-related parameters, which were complemented by macroscopic damage observations.

The proposed experimental program, combining progressively increasing impact-energy levels with a quantitative and qualitative analysis of multiple response parameters, may provide a useful framework for assessing composite configurations intended for naval structures and other applications exposed to accidental low-velocity impact.

## Figures and Tables

**Figure 1:**
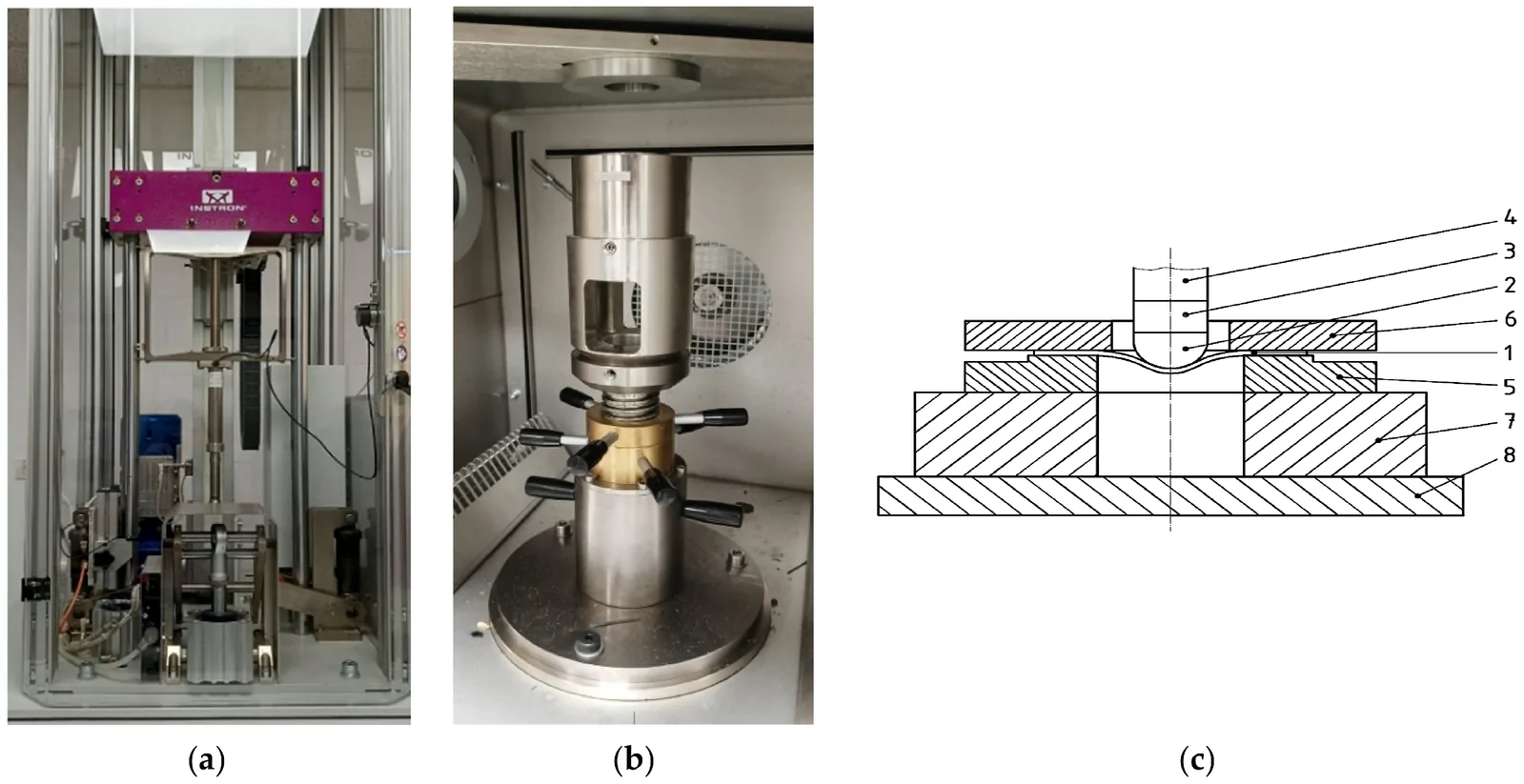
Experimental setup used for the low-velocity impact tests: (**a**) overall view of the Instron CEAST 9350 impact-testing machine; (**b**) close-up view of the hemispherical impactor and pneumatic specimen-clamping system; (**c**) schematic representation of the active test region and specimen clamping arrangement [32]: 1—specimen, 2—hemispherical impactor, 3—force transducer, 4—shaft, 5—specimen support, 6—clamping ring (optional), 7—base frame, 8—acoustic insulation (optional).

**Figure 2:**
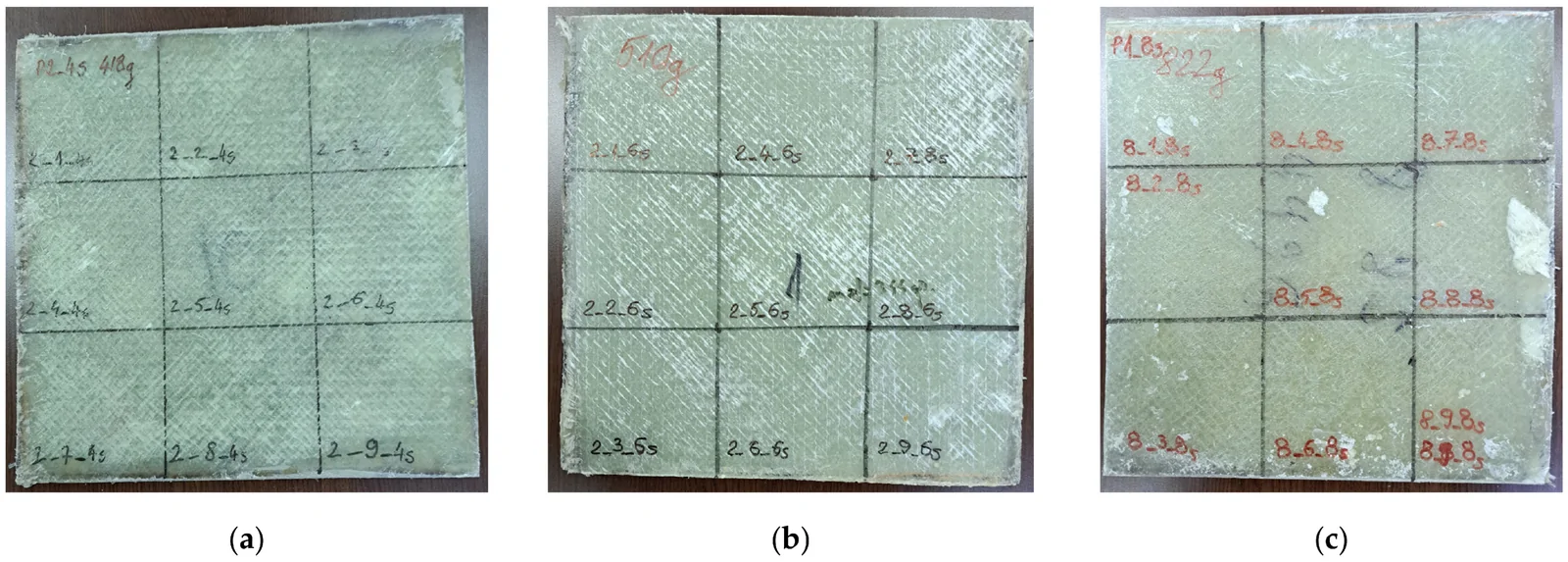
Composite plates made of biaxial glass-fiber fabric and unsaturated polyester resin, marked for cutting into square specimens: (**a**) 4-layer composite; (**b**) 6-layer composite; (**c**) 8-layer composite.

**Figure 3:**
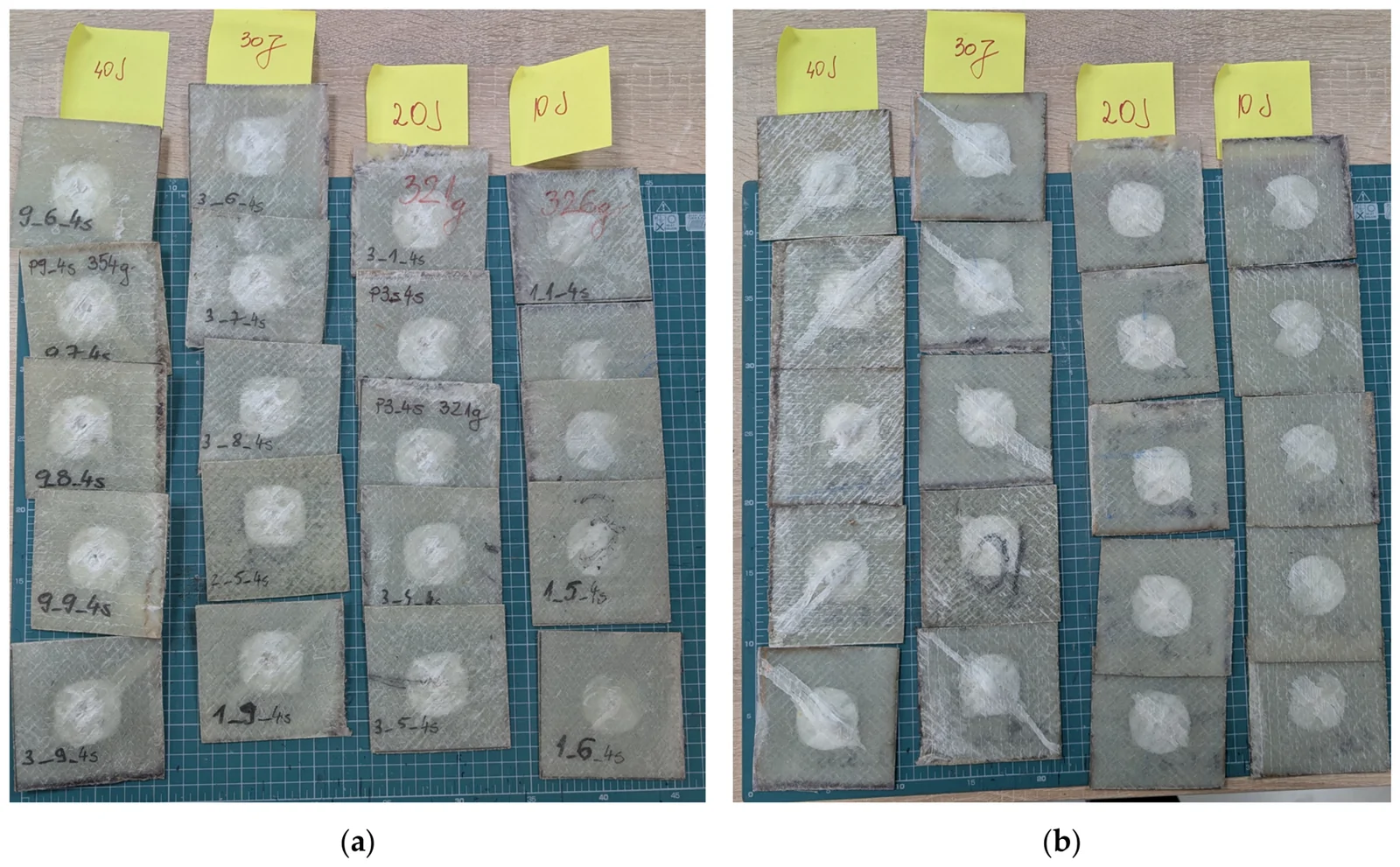
General view of the 4s composite specimens tested at four impact-energy levels: 10 J, 20 J, 30 J, and 40 J. (**a**) Impacted face; (**b**) back face.

**Figure 4:**
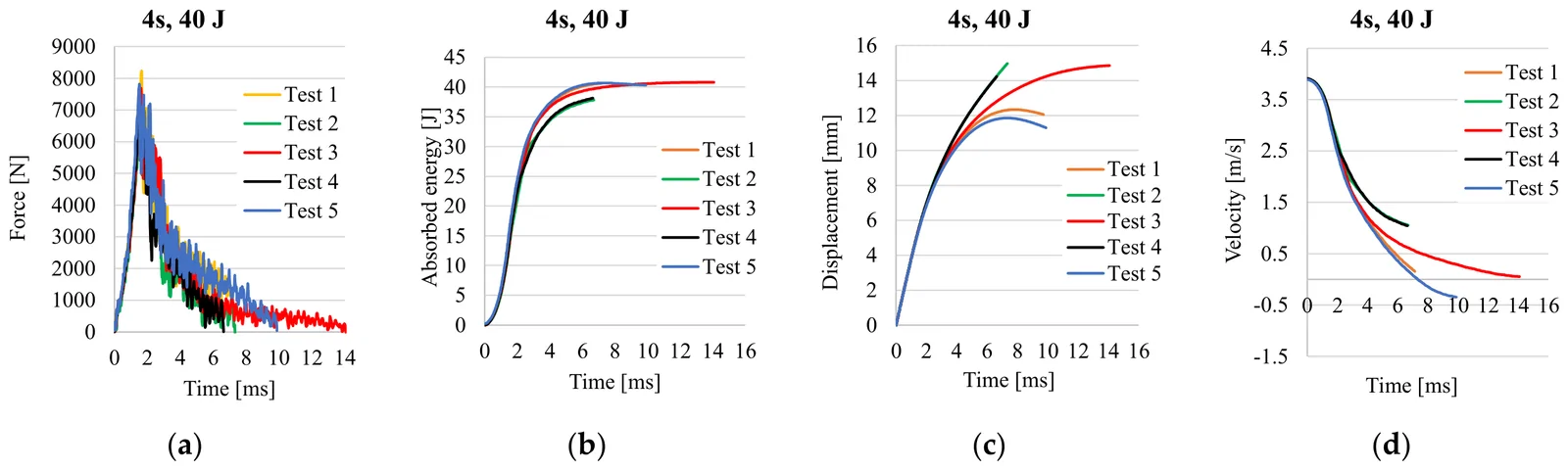
The set of five specimens of the 4s composite tested at an impact energy of 40 J: (**a**) force–time curves; (**b**) absorbed energy–time; (**c**) displacement–time curves; (**d**) velocity–time curves.

**Figure 5:**
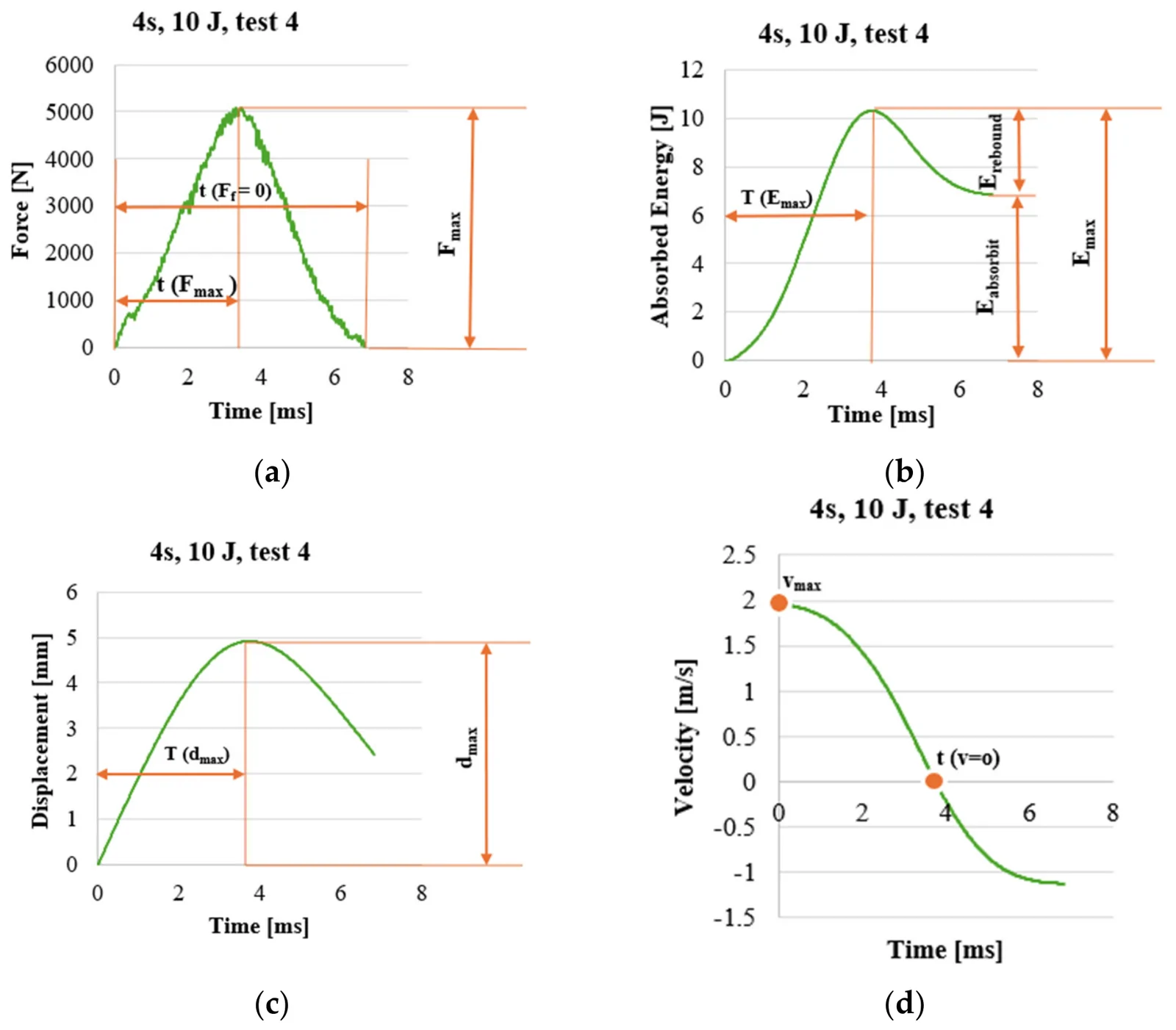
Definition of the selected impact parameters from the recorded histories: (**a**) force–time; (**b**) absorbed energy–time; (**c**) displacement–time; (**d**) velocity–time.

**Figure 6:**
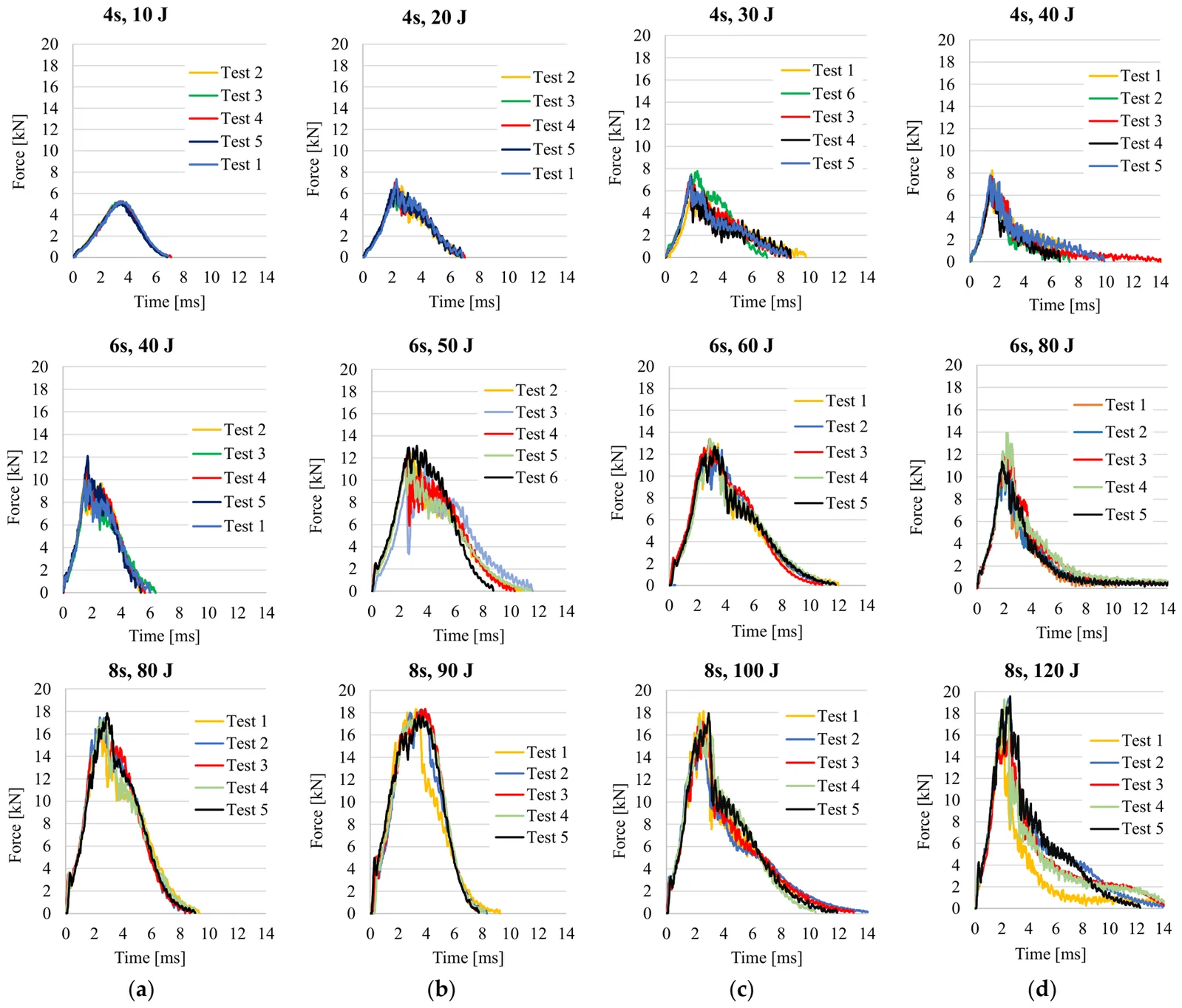
Force–time curves for five replicate tests at four progressively increasing impact-energy levels for each composite configuration: (**a**) lowest investigated impact energy; (**b**,**c**) intermediate impact-energy levels without complete perforation; (**d**) the highest investigated impact-energy level (the transition energy level), at which both partial penetration without complete perforation and complete perforation were observed among the replicate specimens.

**Figure 7:**
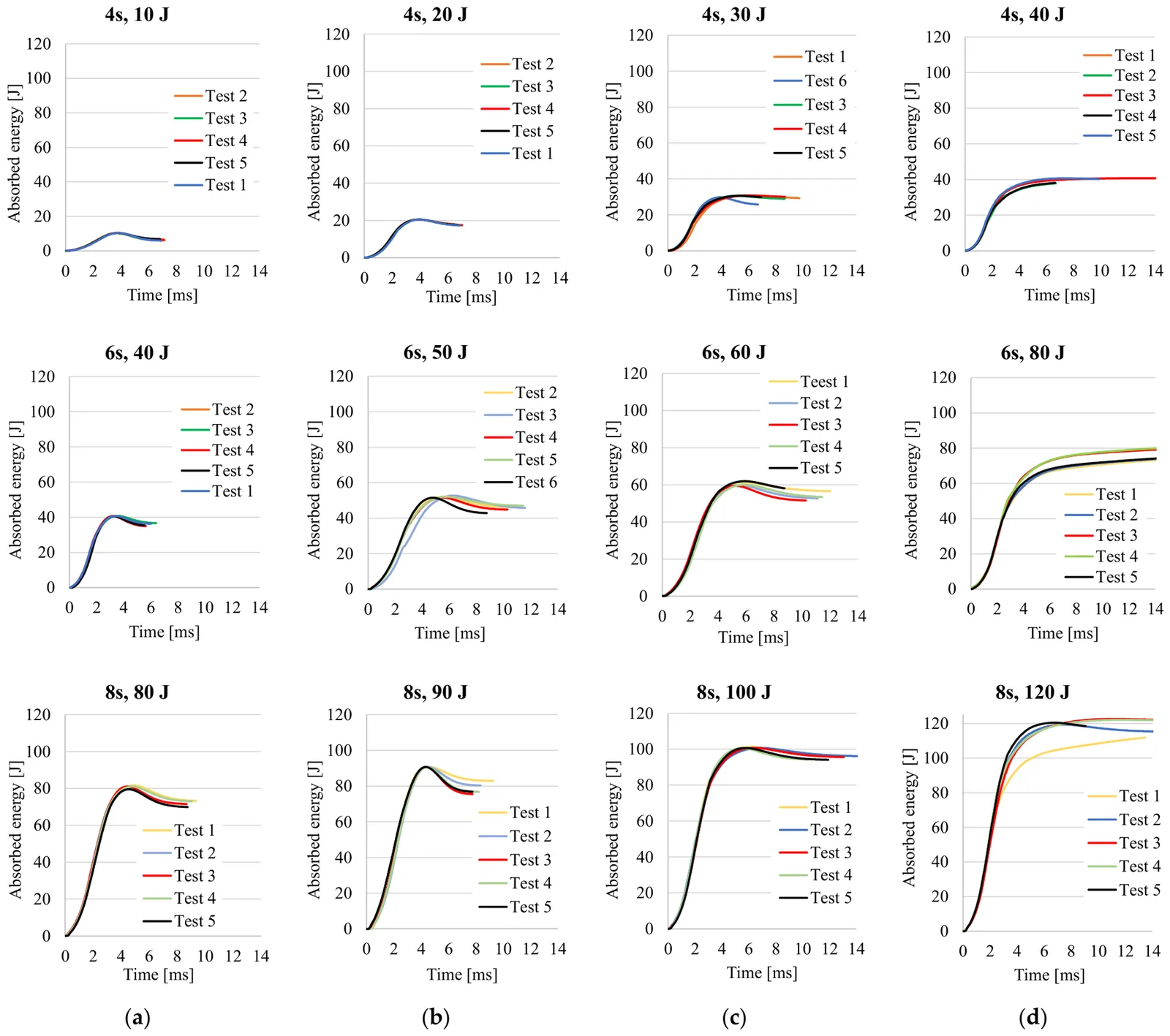
Absorbed energy–time curves for five replicate tests at four progressively increasing impact-energy levels for each composite configuration: (**a**) lowest investigated impact energy; (**b**,**c**) intermediate impact-energy levels without complete perforation; (**d**) the highest investigated impact-energy level (the transition energy level), at which both partial penetration without complete perforation and complete perforation were observed among the replicate specimens.

**Figure 8:**
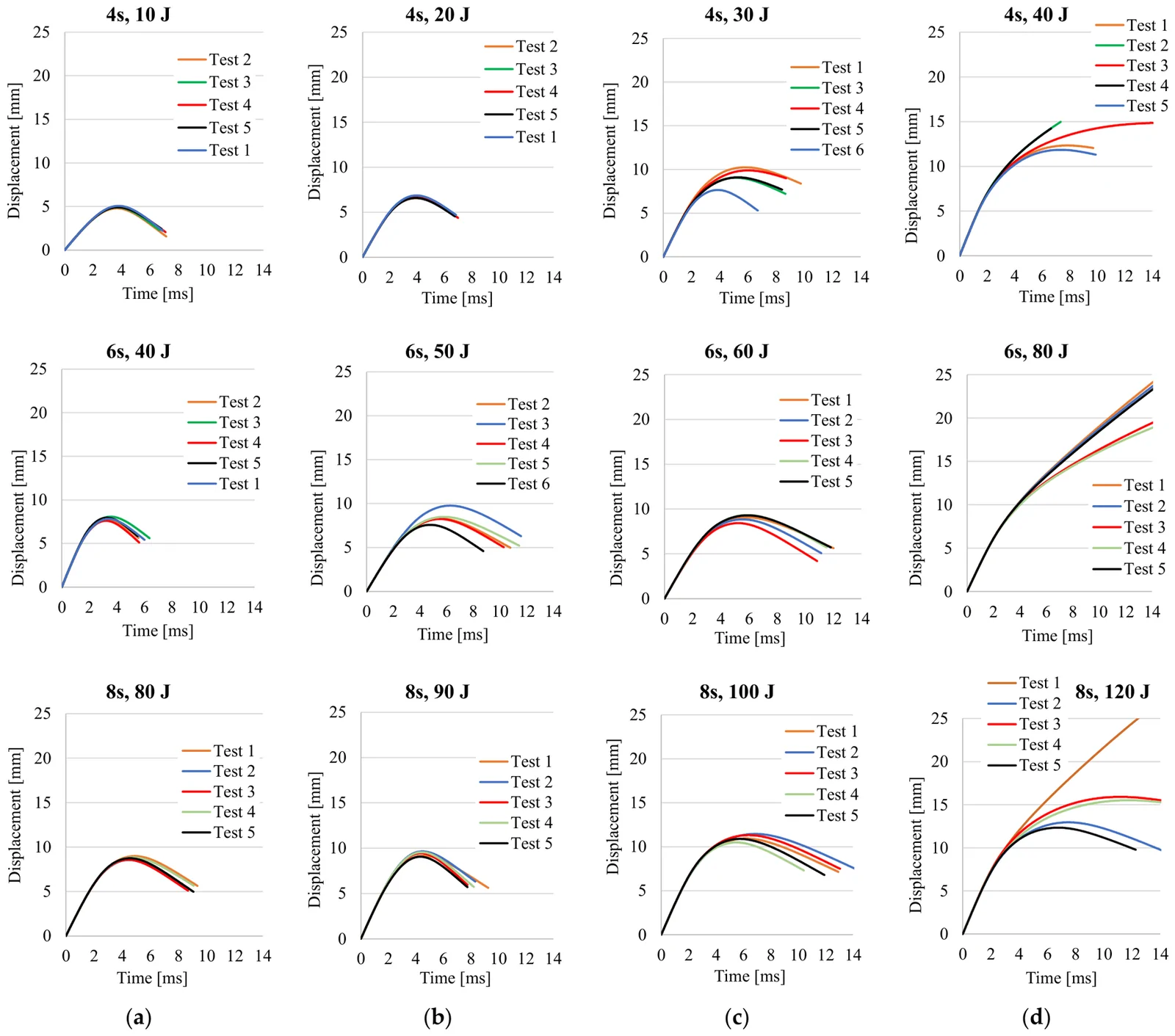
Displacement–time curves for five replicate tests at four progressively increasing impact-energy levels for each composite configuration: (**a**) lowest investigated impact energy; (**b**,**c**) intermediate impact-energy levels without complete perforation; (**d**) the highest investigated impact-energy level (the transition energy level), at which both partial penetration without complete perforation and complete perforation were observed among the replicate specimens.

**Figure 9:**
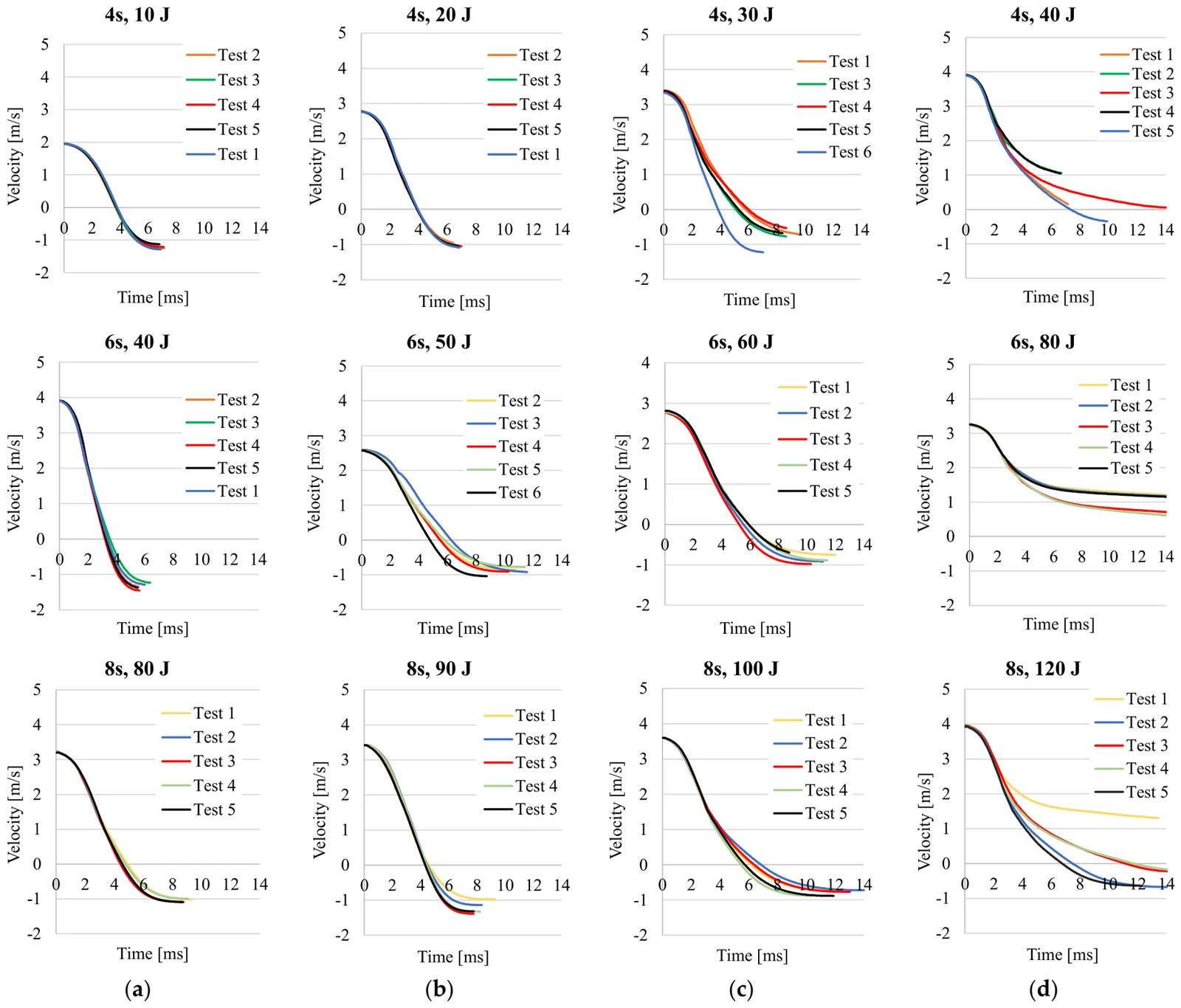
Velocity–time curves for five replicate tests at four progressively increasing impact-energy levels for each composite configuration: (**a**) lowest investigated impact energy; (**b**,**c**) intermediate impact-energy levels without complete perforation; (**d**) the highest investigated impact-energy level (the transition energy level), at which both partial penetration without complete perforation and complete perforation were observed among the replicate specimens.

**Figure 10:**
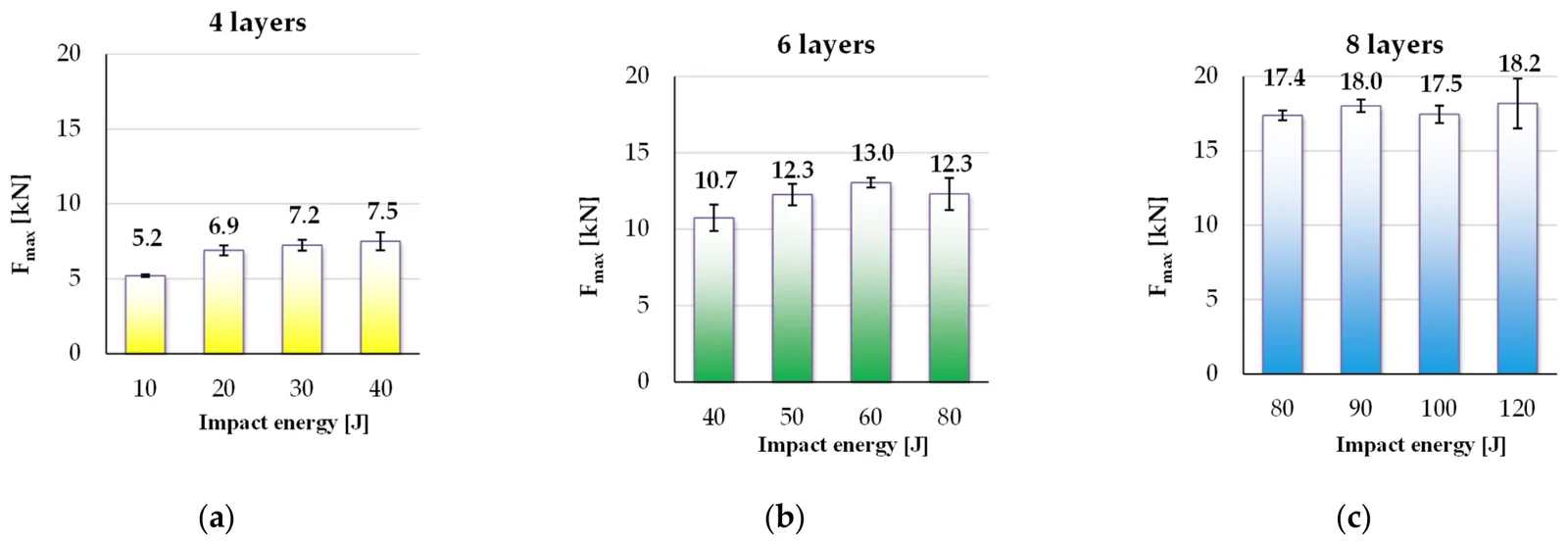
Average values and standard deviations for F_max_ as a function of impact energy for each composite configuration: (**a**) 4-layer composite; (**b**) 6-layer composite; (**c**) 8-layer composite.

**Figure 11:**
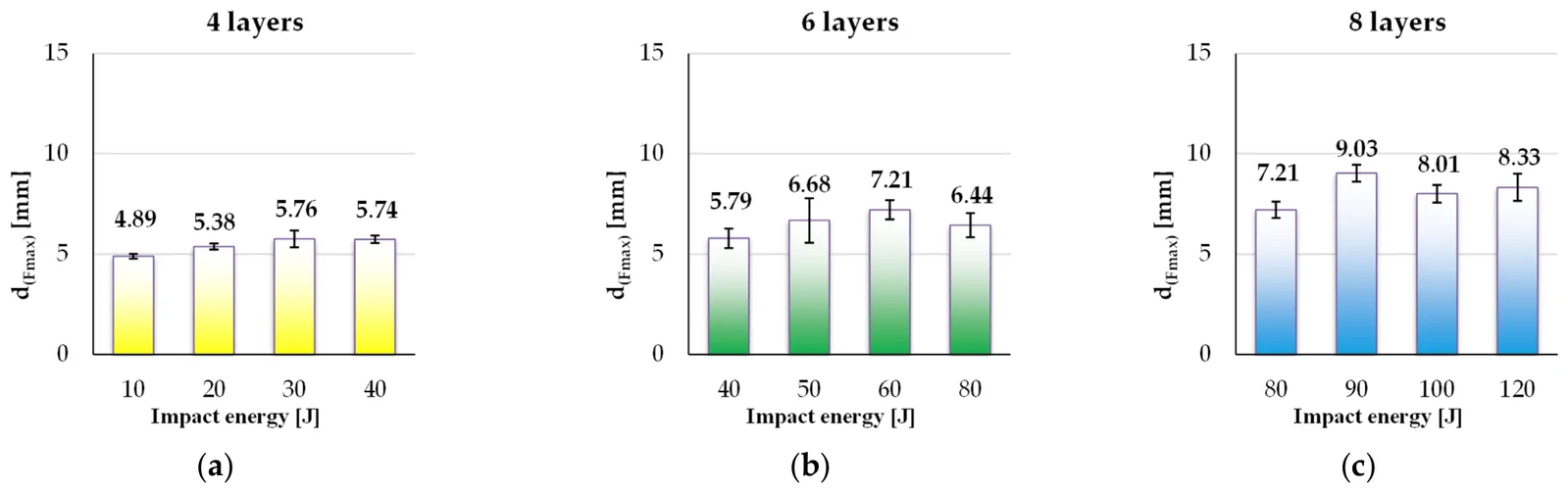
Average values and standard deviations for displacement at F_max_, d_(Fmax)_: (**a**) 4 layers; (**b**) 6 layers; (**c**) 8 layers.

**Figure 12:**
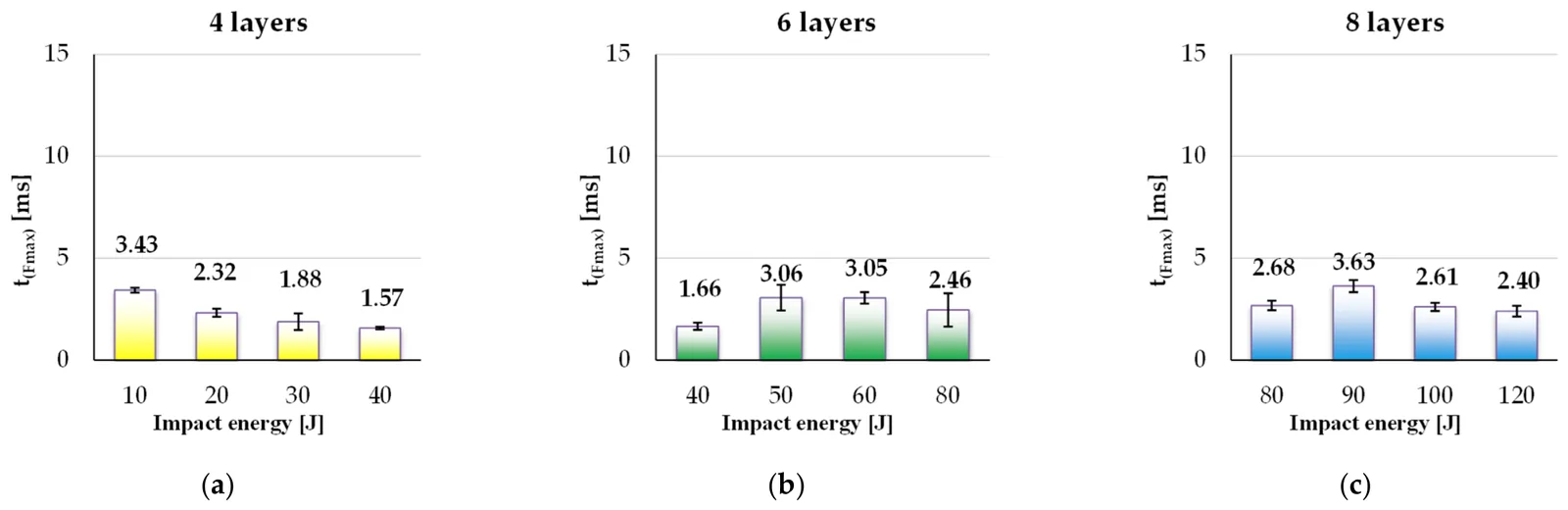
Average values and standard deviations for the time to maximum force, t_(Fmax)_: (**a**) 4 layers; (**b**) 6 layers; (**c**) 8 layers.

**Figure 13:**
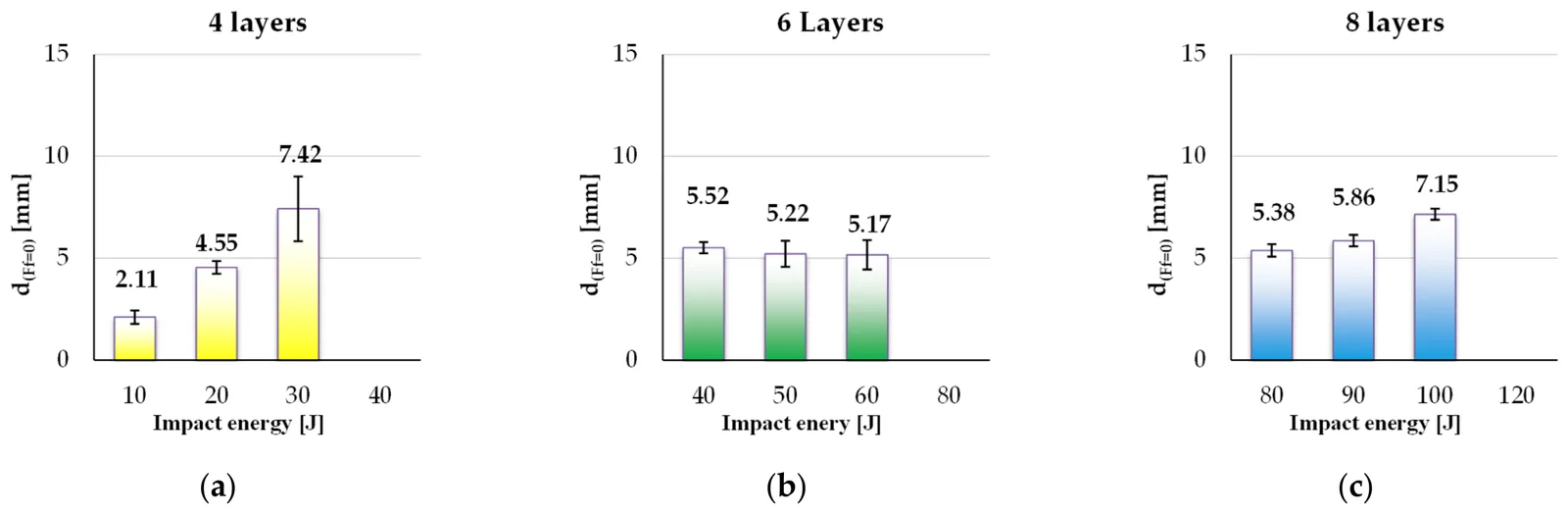
Average values and standard deviations of the impactor displacement at the end of impactor–composite contact, d_(Ff=0)_: (**a**) 4 layers; (**b**) 6 layers; (**c**) 8 layers.

**Figure 14:**
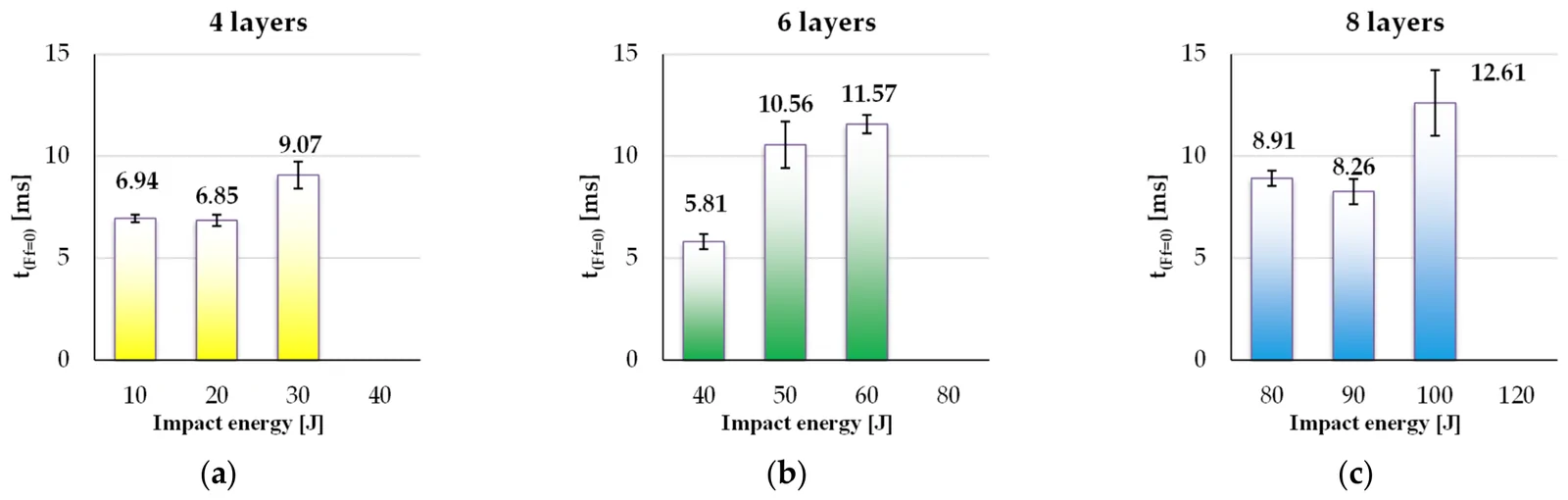
Average values and standard deviations for the time at the end of contact, t_(Ff=0)_, for the three composite configurations: (**a**) 4 layers; (**b**) 6 layers; (**c**) 8 layers.

**Figure 15:**
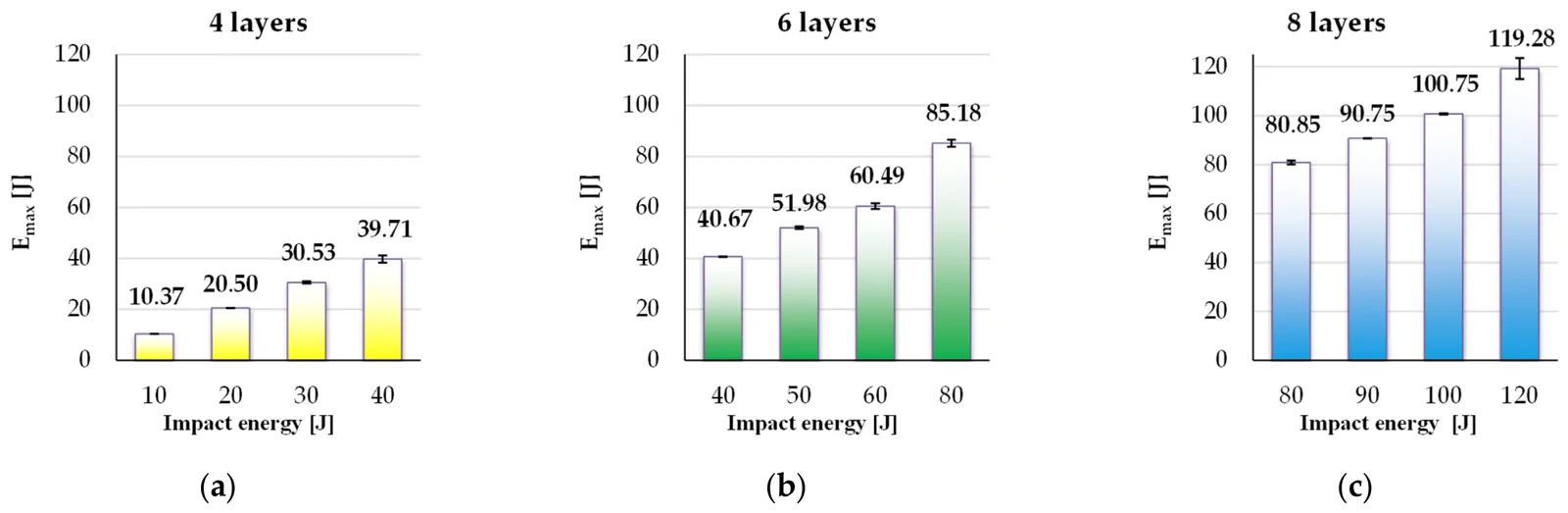
Average values and standard deviations of the maximum recorded energy, E_max,_ for the three composite configurations: (**a**) 4 layers; (**b**) 6 layers; (**c**) 8 layers.

**Figure 16:**
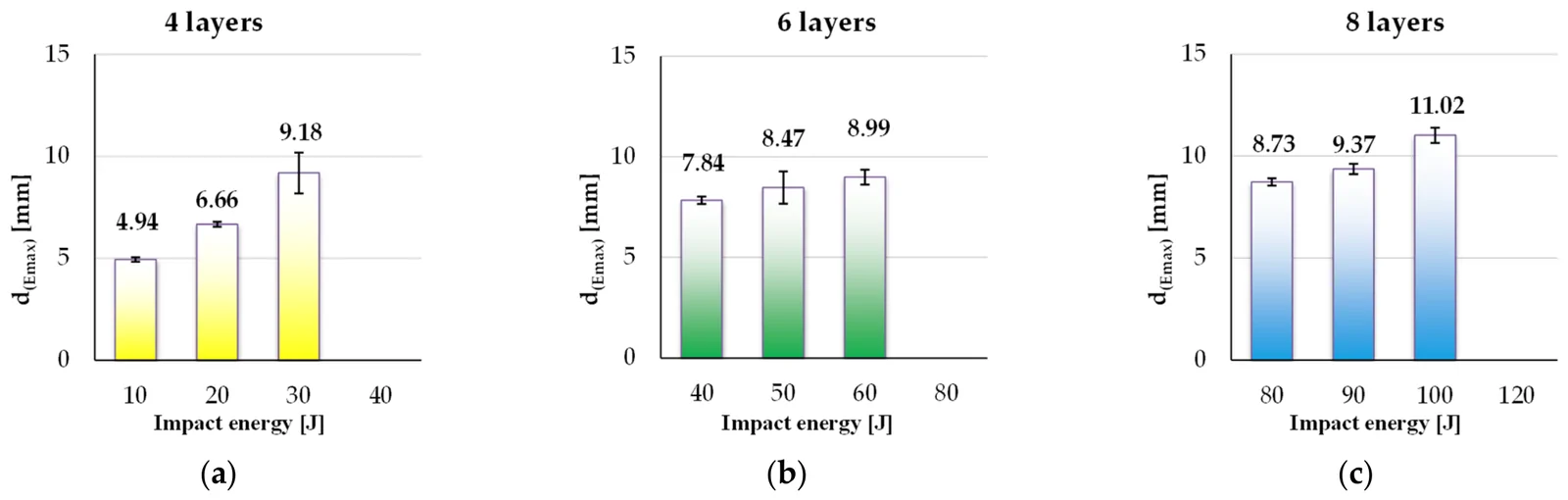
Average values and standard deviations of the displacement at maximum recorded energy, d_(Emax),_ for the three composite configurations: (**a**) 4 layers; (**b**) 6 layers; (**c**) 8 layers.

**Figure 17:**
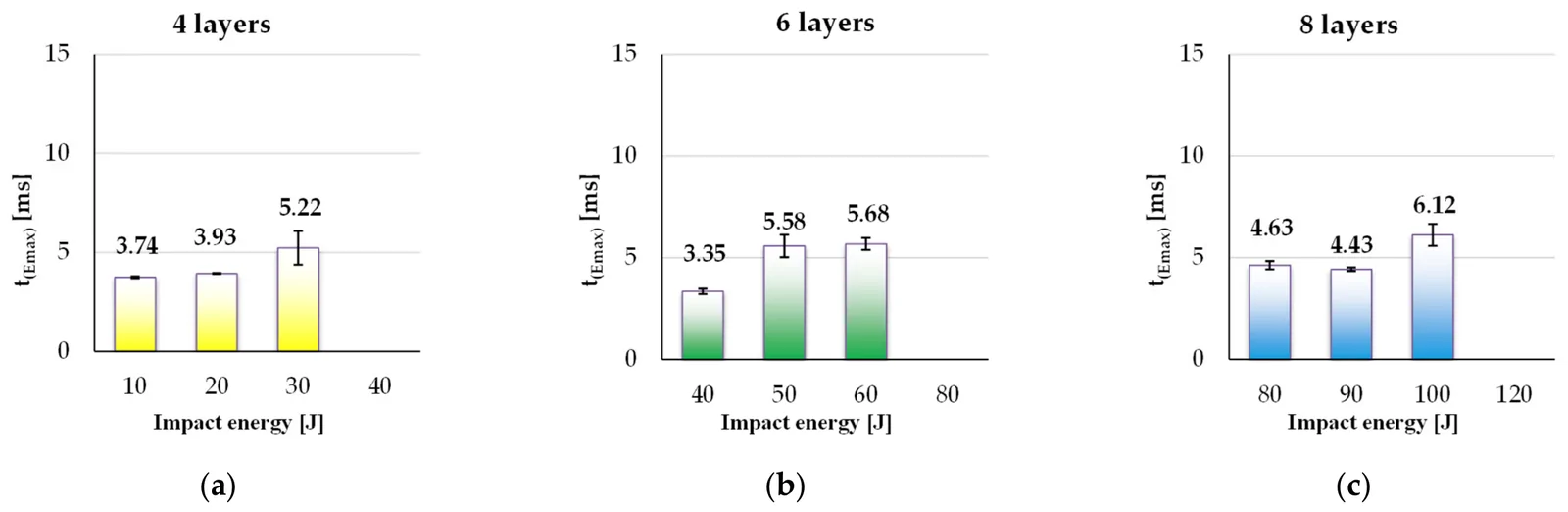
Average values and standard deviations of the time to maximum recorded energy, t_(Emax)_, for the three composite configurations: (**a**) 4 layers; (**b**) 6 layers; (**c**) 8 layers.

**Figure 18:**
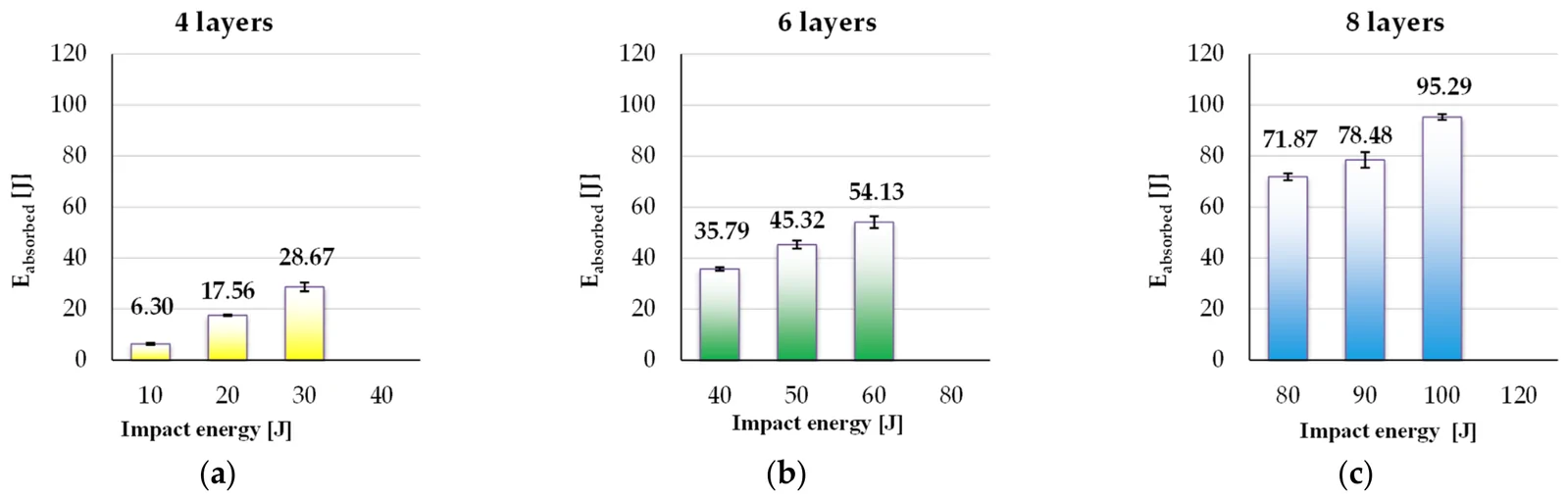
Average values and standard deviations of the absorbed energy, E_absorbed_, for the three composite configurations: (**a**) 4 layers; (**b**) 6 layers; (**c**) 8 layers.

**Figure 19:**
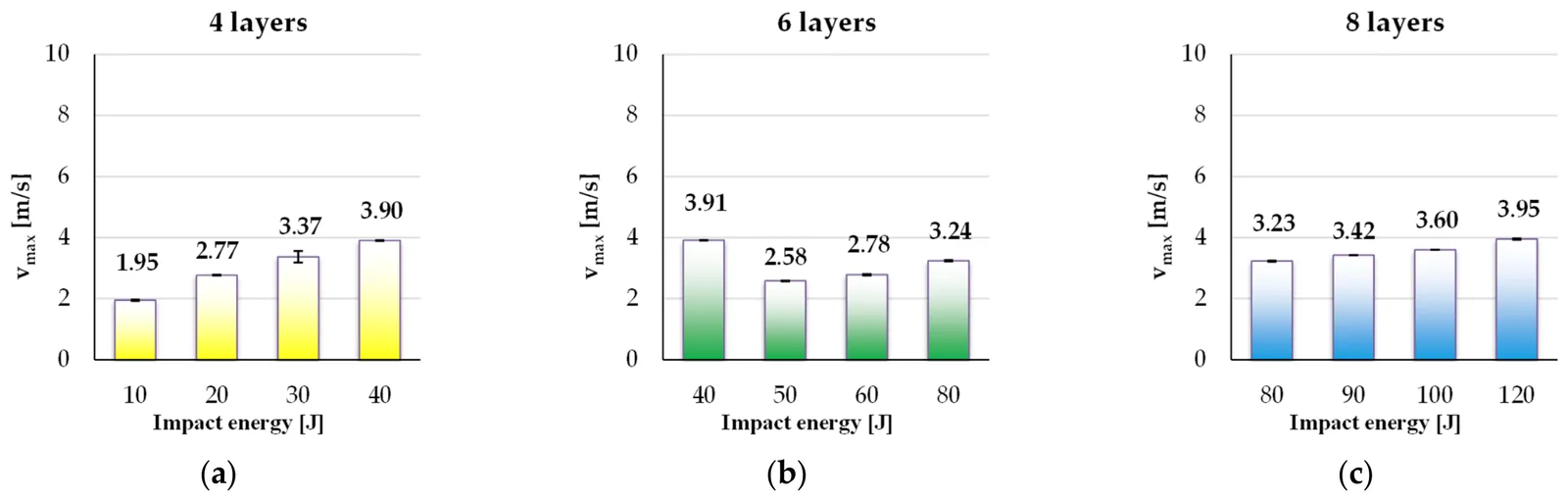
Average values and standard deviations of the maximum impactor velocity, v_max_, for the three composite configurations: (**a**) 4 layers; (**b**) 6 layers; (**c**) 8 layers.

**Figure 20:**
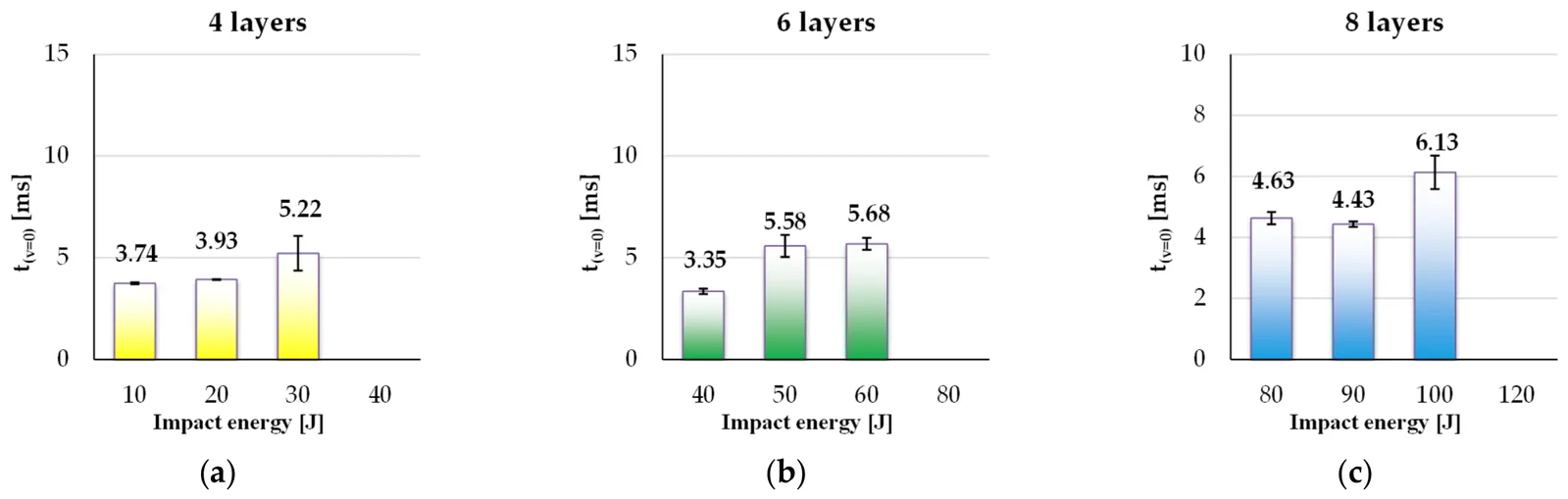
Average values and standard deviations of the time to zero impactor velocity, t_(v=0)_, for the three composite configurations: (**a**) 4 layers; (**b**) 6 layers; (**c**) 8 layers.

**Figure 21:**
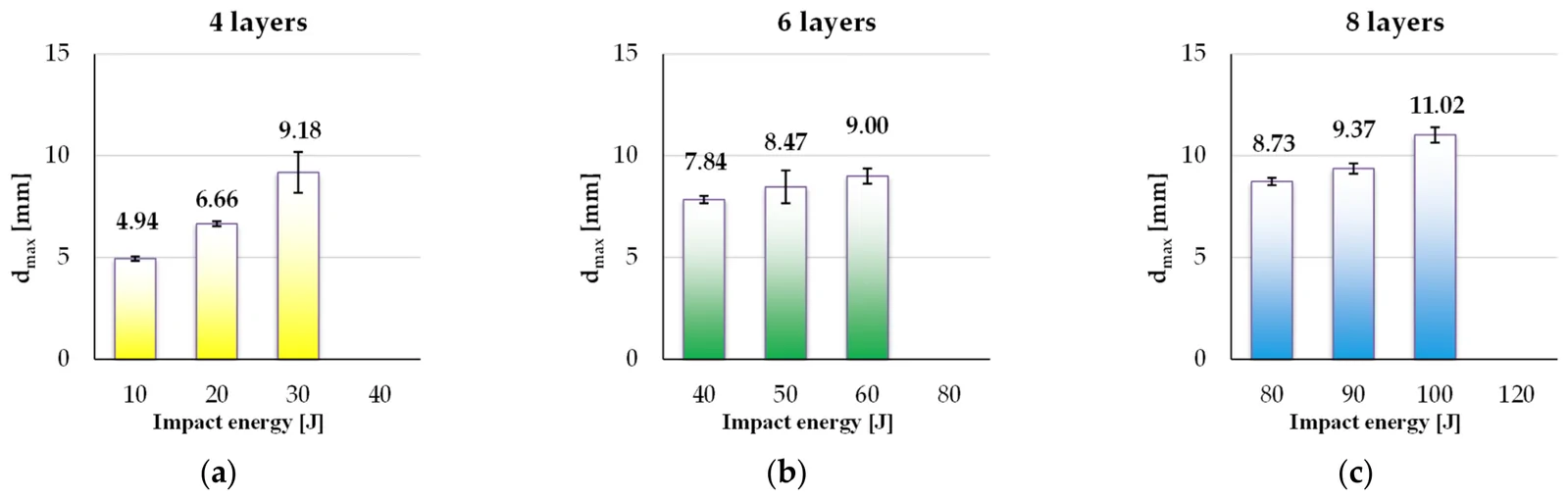
Average values and standard deviations of the maximum displacement, d_max_, for the three composite configurations: (**a**) 4 layers; (**b**) 6 layers; (**c**) 8 layers.

**Figure 22:**
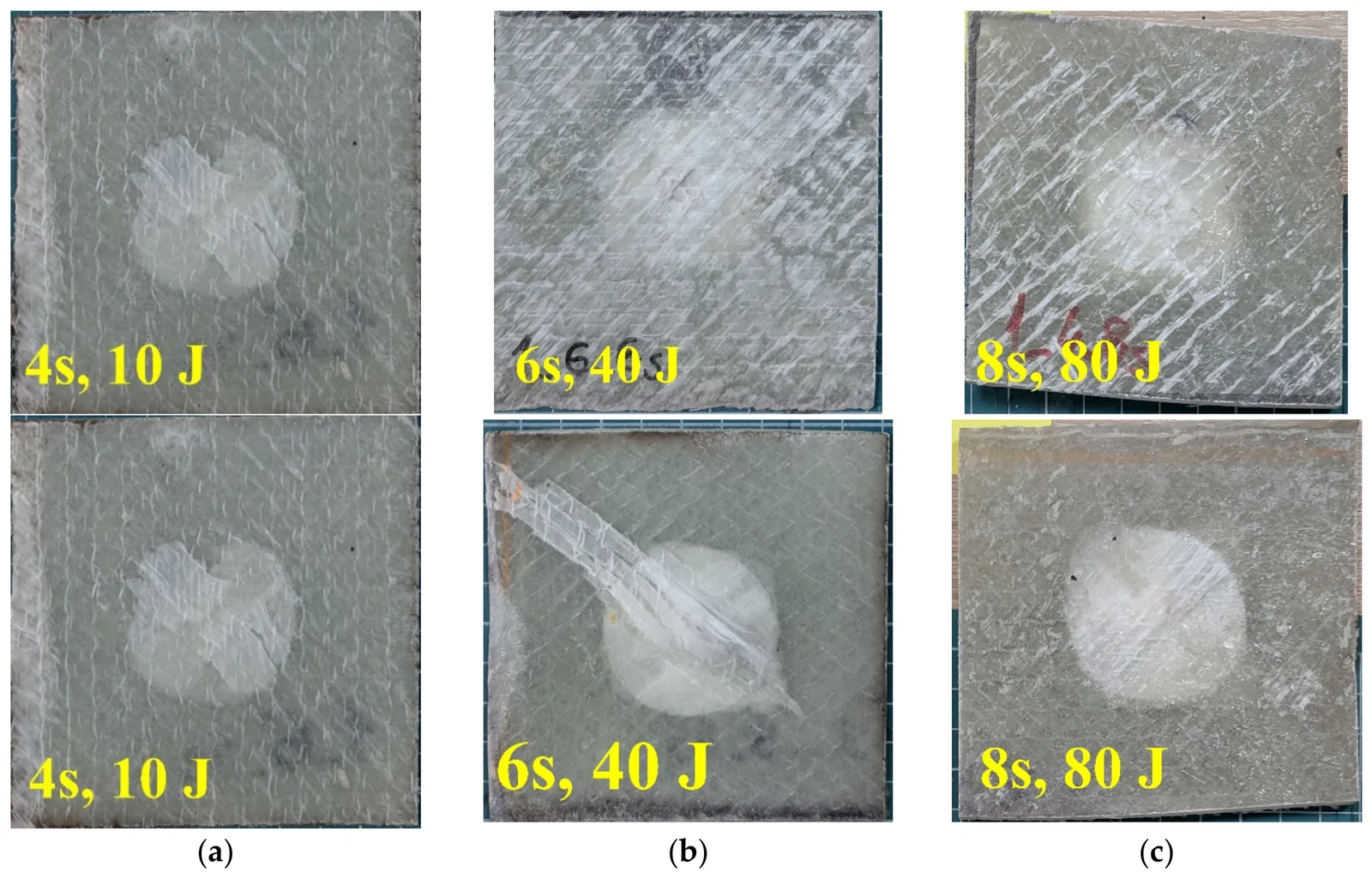
Representative specimens at the lowest investigated impact-energy level. Upper row: impacted face; lower row: back face. (**a**) 4s, 10 J; (**b**) 6s, 40 J; (**c**) 8s, 80 J.

**Figure 23:**
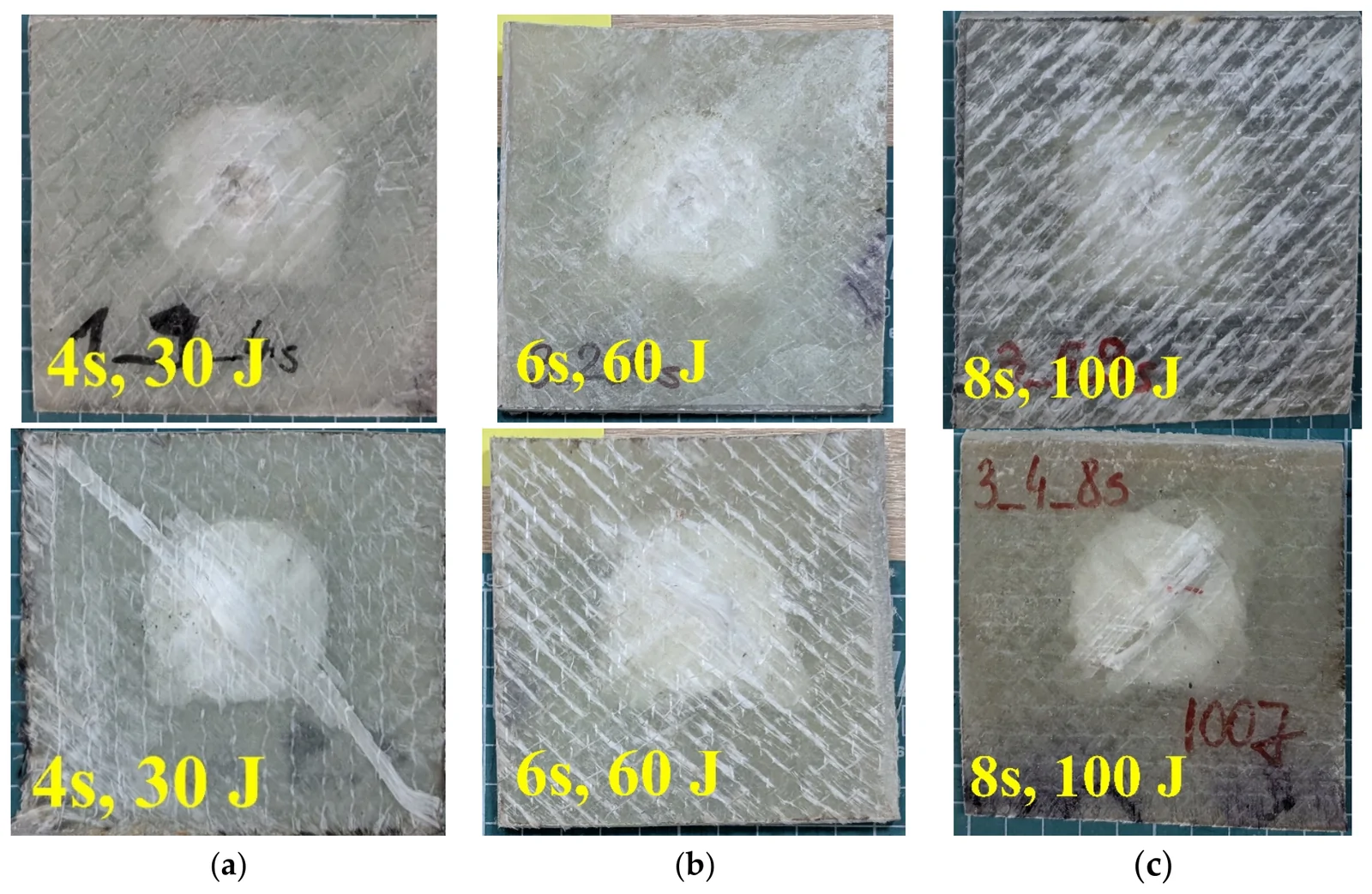
Representative specimens tested at the highest investigated impact-energy level without complete perforation. Upper row: impacted face; lower row: back face. (**a**) 4s, 30 J; (**b**) 6s, 60 J; (**c**) 8s, 100 J.

**Figure 24:**
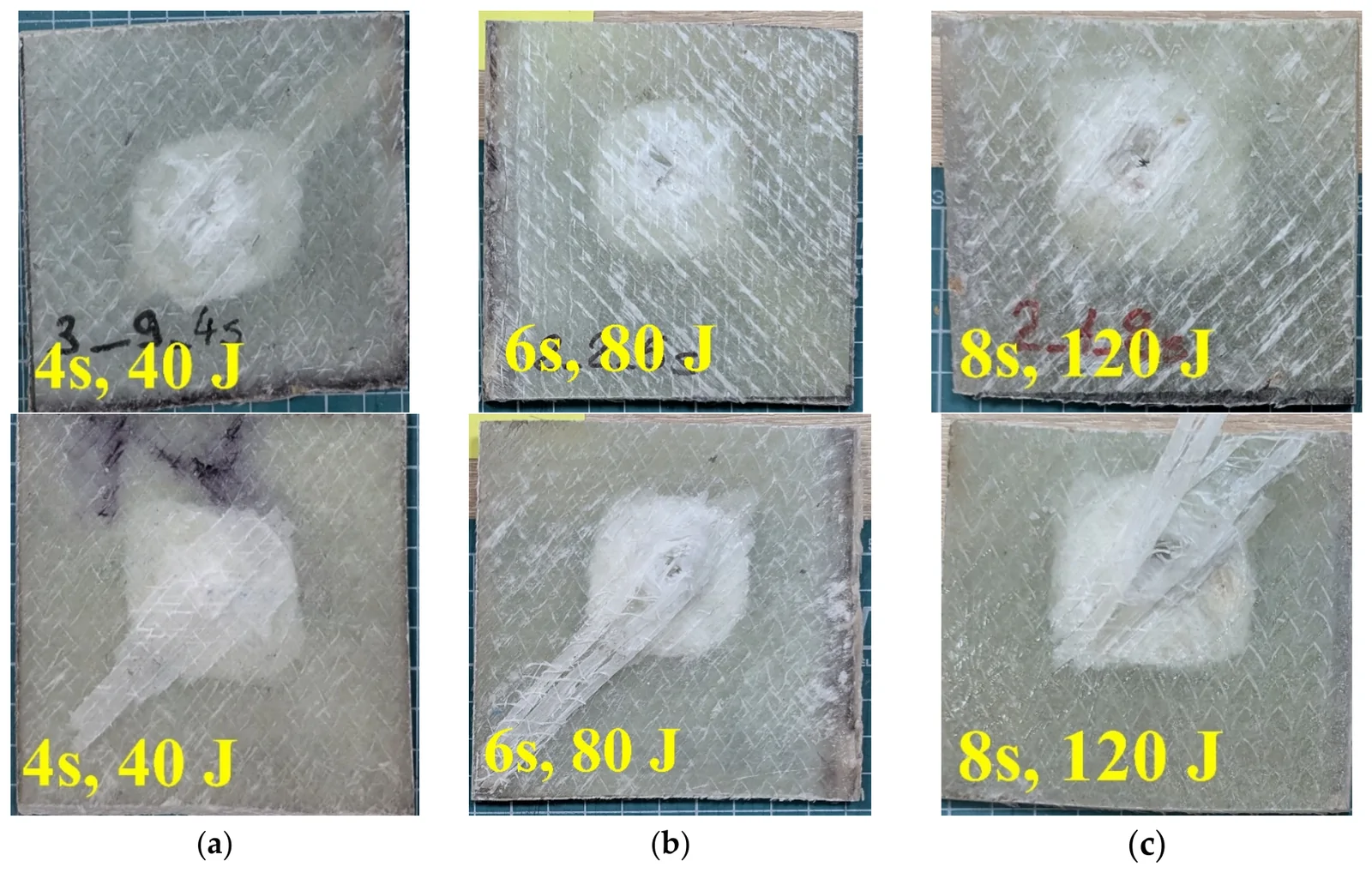
Representative specimens exhibiting complete perforation at the transition impact-energy level. Upper row: impacted face; lower row: back face. (**a**) 4s, 40 J; (**b**) 6s, 80 J; (**c**) 8s, 120 J.

**Table 1:** Average mass and thickness characteristics of the tested specimens.

Composite	Specimen Mass, m_c set_ [g]		Theoretical Mass of the Specimen Fabric, m_f set_ [g]		Calculated Resin Mass, m_r set_ [g]		Fiber Mass Ratio, *R*_(*mf*/*c*)*set*_ [-]		Thickness of the Specimens, h_set_ [mm]	
	*	CV [%]	*	CV [%]	*	CV [%]	*	CV [%]	*	CV [%]
4s	35.55 ± 2.28	6.41	24.26 ± 0.56	2.31	11.29 ± 2.21	19.57	0.68 ± 0.04	5.88	2.22 ± 0.24	10.81
6s	59.75 ± 6.75	11.30	36.12 ± 1.85	5.12	23.63 ± 5.99	25.35	0.61 ± 0.06	9.84	3.78 ± 0.43	11.38
8s	84.80 ± 9.74	11.49	49.81 ± 1.73	3.47	34.99 ± 9.32	26.64	0.59 ± 0.07	11.86	5.39 ± 0.48	8.91

* Values are reported as average ± standard deviation for *N* = 20 specimens per composite configuration.

**Table 2:** Tests carried out for this study.

Composite	Nominal Impact Energy [J]									
	10	20	30	40	50	60	80	90	100	120
4s										
6s										
8s										

**Table 3:** Impact-test conditions for the investigated composite configurations.

Composite	E_nominal_ [J]	Drop Mass [kg]	Impact Velocity * [m/s]	Drop Height [mm]	Effective Impact Energy * [J]
4s	10	5.277	1.953 ± 0.006	193	10.067 ± 0.060
	20	5.277	2.767 ± 0.006	386	20.199 ± 0.087
	30	5.277	3.373 ± 0.024	580	30.021 ± 0.422
	40	5.277	3.896 ± 0.008	773	40.057 ± 0.160
6s	40	5.277	3.909 ± 0.008	773	40.326 ± 0.168
	50	15.277	2.579 ± 0.008	334	50.804 ± 0.299
	60	15.277	2.783 ± 0.024	401	59.151 ± 1.041
	80	15.277	3.244 ± 0.017	534	80.408 ± 0.841
8s	80	15.277	3.226 ± 0.015	534	79.518 ± 0.738
	90	15.277	3.422 ± 0.001	601	89.427 ± 0.040
	100	15.277	3.602 ± 0.004	667	99.127 ± 0.245
	120	15.277	3.953 ± 0.022	801	119.335 ± 1.302

* Values are reported as average ± standard deviation for each set of five tested specimens for each composite configuration and set of test conditions.

**Table 4:** Diagnostic assessment of the selected impact-response parameters.

Para- Meter	Main Observed Behavior	Sensitivity to Test Conditions	Diagnostic Value	Physical Interpretation/Limitations
F_max_	Increases with thickness and energy; tends to plateau near the critical regime	Low–moderate	Moderate	Maximum load-bearing response; alone does not identify perforation
t_(Fmax)_	Non-monotonic; generally decreases at the critical energy	Moderate	Moderate	Indicates how rapidly peak load is reached; requires interpretation with other histories
d_(Fmax)_	Limited, non-monotonic variation with energy	Moderate	Low–moderate	Deformation at peak force; occurs relatively early in the impact event
d_(Ff=0)_	Generally increases with damage severity before perforation	Moderate	Moderate	Apparent residual displacement; meaningful only when the impactor is arrested
t_(Ff=0)_	Non-monotonic	Moderate	Low–moderate	Characterizes the end of contact in rebound tests; not consistently defined after perforation
E_absorbed_	Increases markedly with energy and number of fabric layers before perforation	Moderate	High	Energy dissipated by deformation and damage; after perforation also includes path formation and friction
t_(Emax)_	Occurs after t_(Fmax)_; tends to increase near the upper non-perforating level	Moderate	Moderate	Reflects continued energy transfer after peak force
d_(Emax)_	Increases with impact severity, especially for 4s	Low–moderate	Moderate–high	Displacement at maximum energy transfer; comparison should remain within the same impact regime
v_max_	Governed mainly by mass–drop-height combination	High	Very low	Test-control parameter; small scatter indicates repeatable impact conditions
t_(v=0)_	Strongly affected by mass–velocity combination	High	Moderate *	Identifies impactor arrest; meaningful only before complete perforation
d_max_	Increases consistently toward the highest non-perforating energy	Low–moderate	High	Strong indicator of increasing impact severity before perforation

* within the non-perforating regime.

## Data Availability

Data are contained within the article.
